# Unravelling the human taste receptor interactome: machine learning and molecular modelling insights into protein-protein interactions

**DOI:** 10.1038/s41538-025-00478-9

**Published:** 2025-07-01

**Authors:** Harry Zaverdas, Filip Stojceski, Rocío Romero-Zaliz, Lampros Androutsos, Pantelis Makrygiannis, Lorenzo Pallante, Vanessa Martos, Gianvito Grasso, Marco A. Deriu, Konstantinos Theofilatos, Seferina Mavroudi

**Affiliations:** 1 InSyBio PC, Patras, Greece; 2 Department of Medicine, School of Health Sciences, University of Patras, Patras, Greece; 3 Dalle Molle Institute for Artificial Intelligence USI-SUPSI, Polo universitario Lugano—Campus Est, Lugano, Switzerland; 4 Department of Computer Science and AI, Research Center in Information and Communication Technologies (CITIC), Andalusian Research Institute on Data Science and Computational Intelligence (DaSCI), University of Granada, Granada, Spain; 5 PolitoBIOMedLab, Department of Mechanical and Aerospace Engineering, Politecnico di Torino, Torino, Italy; 6 Department of Plant Physiology, University of Granada, Granada, Spain; 7 Institute of Biotechnology (IBT-UGR), University of Granada, Granada, Spain; 8 School of Cardiovascular and Metabolic Medicine & Sciences, King’s College London, London, UK; 9 Department of Nursing, School of Health Rehabilitation Sciences, University of Patras, Patras, Greece

**Keywords:** Predictive markers, G protein-coupled receptors, Molecular modelling, Biochemical networks, Regulatory networks

## Abstract

The understanding of the molecular mechanisms that drive taste perception can have broad implications for public health. This study aims to expand the understanding of taste receptor-associated molecular pathways by resolving the taste receptor interactome. To this end, we propose a comprehensive machine learning approach to accurately predict and quantify protein-protein interactions using an ensemble evolutionary algorithm. 1,647,374 positive and 894,213 negative experimentally verified protein-protein interactions were mined and characterized using 61 functional orthology, sequence, co-expression and structural features. The binary classifier significantly improved the accuracy of existing methods, reconstructing the full taste receptor interactome and was combined with a regressor that estimates the binding strength of positive interactions. Molecular dynamics investigation of top-scoring protein-protein interactions verified novel interactions of TAS2R41. The reconstructed TR interactome can catalyze the study of molecular pathophysiological mechanisms related to taste, the development of flavorsome nutrient-dense food products and the identification of personalized nutrition markers.

## Introduction

Proteins are versatile macromolecules that have essential roles in organisms^1,2^. They perform a wide range of tasks, including those connected to storage, transport, enzymatic reactions, and structural functions in cell membranes^3–5^. Protein–Protein Interactions (PPIs) play a pivotal role in a wide spectrum of cellular processes, including taste perception^6^, signal transduction^7^, immunological responses^8^, DNA transcription^9^, DNA replication^10^, enzymatic reactions^11^, cell adhesion^12^, metabolic pathways^13^, and much more. The role of PPIs is particularly significant in the context of taste receptors (TRs), which are responsible for detecting various taste modalities^14^. These receptors interact with specific proteins to initiate signaling pathways that influence dietary choices and preferences^15^. Understanding these interactions can shed light on how taste perception is linked to nutritional behavior and how it may be altered in conditions such as obesity, diabetes, and other nutrition-related disorders^16^. The main objective of the present manuscript was to fully resolve the interactome of TR, but to achieve this, more accurate and efficient methods were needed for the computational prediction of PPIs and the estimation of their binding affinity.

High-throughput technologies, including techniques like yeast two-hybrid (Y2H) screens^17^ and mass spectrometry methods^18^, have led to the accumulation of data on PPIs from various experiments, resulting in the establishment of multiple databases. Computational analysis and classification of PPIs have emerged as a crucial and efficient auxiliary tool for addressing open challenges in the post-genomic era.

In recent years, there has been a significant surge in the development of computational methods for predicting PPIs^19,20^. These methods can be broadly classified into two categories: classic machine learning (ML)^21^ and deep learning (DL)^22^ algorithms. Various traditional ML techniques, such as Support Vector Machine (SVM), Naive Bayes (NB), K-Nearest Neighbor (KNN), and Random Forests (RF), have been employed to predict PPIs^23^. SVM has emerged as one of the most extensively utilized ML-based approaches^24–29^. These ML methods have utilized features directly related to the protein’s amino acid sequence for PPI prediction, such as amino acid composition (AAC)^30,31^, Pseudo-AAC^32^, dipeptide composition^33^, and tripeptide composition^34^. Although many ML algorithms have been developed for PPI prediction, their classification performance can still be improved, and the challenge of calculating the binding strength occurring between proteins involved in PPI persists.

To bridge this gap, experimental procedures to collect dissociation constants (*K*_*d*_) between proteins have become fundamental in providing reliable data for PPI studies. Techniques such as surface plasmon resonance (SPR)^35^, isothermal titration calorimetry (ITC)^36^, Fluorescence Resonance Energy Transfer (FRET)^37^, and Biolayer interferometry (BLI)^38^ are widely used to obtain precise measurements of binding affinities. These methods offer high accuracy and reproducibility, making them the gold standard for generating binding affinity data. Recently, advances in ML algorithms have enabled the development of novel models to predict binding affinities, complementing and extending experimental techniques^39,40^. These advancements demonstrate the growing potential of ML in predicting binding affinities, providing a valuable complement to experimental data, however, their time and computational resources requirements make them unsuitable for screening and studying the overall interactome of an organism. By incorporating more efficient computational models, researchers can gain deeper insights into protein interactions.

This study focuses on PPIs within the context of the TR interactome to gain insights into the associated downstream molecular pathways^41^. TRs are highly specialized proteins responsible for detecting chemicals in the ingested foods and recognizing five primary tastes, including sweet, umami, bitter, sour, and salty^42,43^. As a result of their undeniable achievements, molecular modeling methodologies are progressively gaining popularity in the fields of food science and taste perception^6,44^. There are several structure-based molecular modeling methods, such as molecular docking and molecular dynamics (MD), that have been widely applied for studying TRs^6,45–47^. In this study, we propose a comprehensive approach to predict and quantify PPIs with a focus on elucidating TR-associated molecular pathways. The selected methodology integrates ML algorithms with a fine-tuned evolutionary optimization algorithm (EOA) to accurately predict PPIs. To train and test models, we have put together a fully annotated dataset of experimentally verified positive and negative PPIs with 61 features. These features contain functional similarity, orthologous interactions in other organisms, PPI data mined from other databases, and sequential, co-expression, and structural information for each protein pair. In a similar way, we have gathered experimental data on protein–protein binding affinities and trained an ML-driven computational model for estimating the binding strength of PPIs. In addition, our investigation led to the discovery of a novel interaction between TAS2R41 and CHMP4A proteins, identified through MD simulations of a selected PPI among the top-scored PPIs predicted by the EOA model. These findings highlight the efficacy of this multi-disciplinary approach in uncovering biologically relevant interactions and shed light on the potential molecular mechanisms underlying taste perception.

## Results

**Fig. 1:**
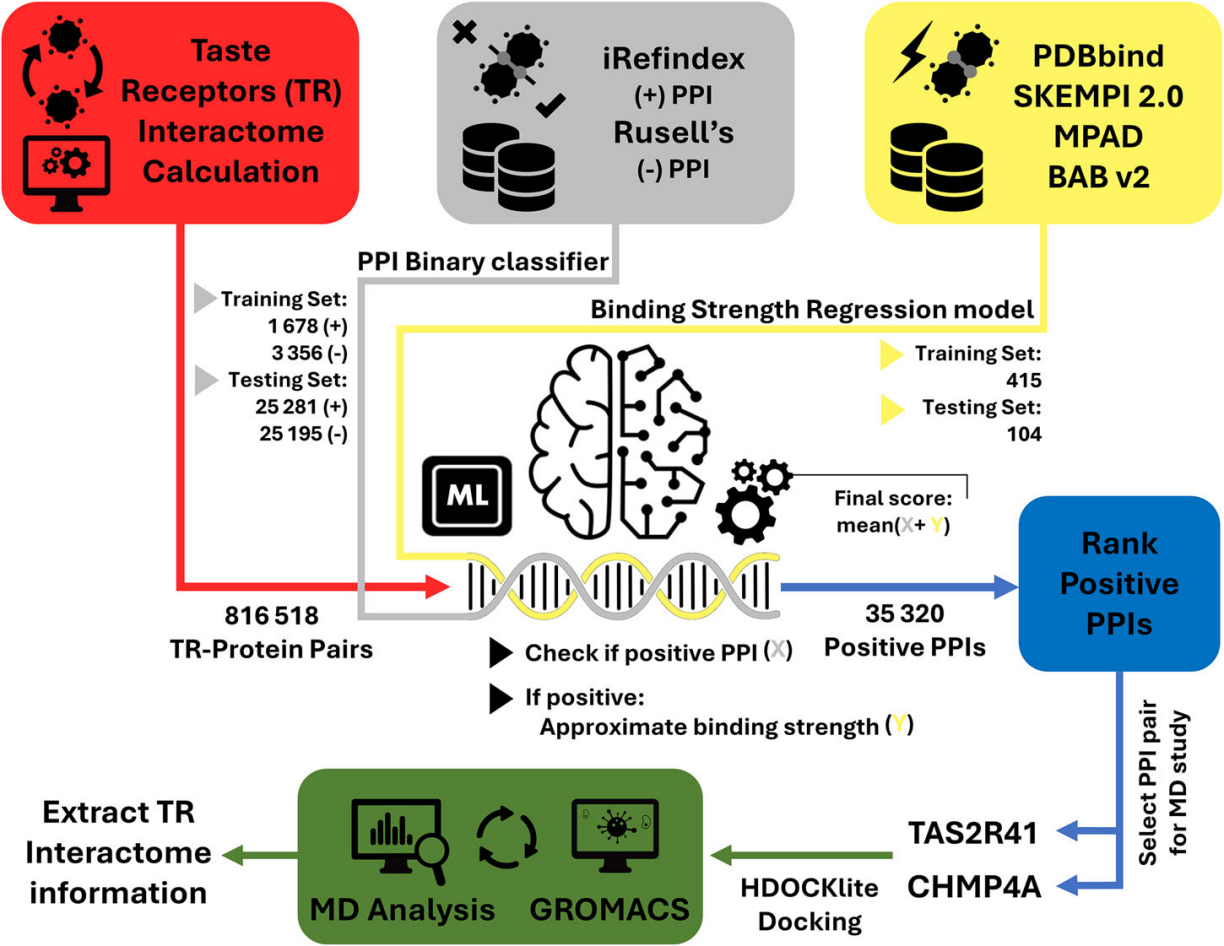
Flowchart of the proposed method. Red colors are used for taste receptors (TR) interactome, gray colors are used for the PPI binary classifier ML model, yellow colors are used for the binding strength regression model, blue color is used for the selection of the PPI pair for the Molecular Dynamics (MD) study, and green colors are used for MD modeling.

The first goal was to design both a binary classifier and a regressor to identify new interactions between TRs and other proteins. The classifier is used for identifying the positive TR interactions from the vastness of the TR interactome, while the regressor provides a computational affinity measurement for these positive interactions. In conjunction with the classifier’s probability, the predicted affinity was used for ranking and identifying the most probable TR interactions in order to be further validated with techniques such as MD analysis. In detail, two methodologies were implemented for the creation and evaluation of the binary classifier for PPI prediction, using benchmark machine learning (BML) techniques and an EOA. For the creation of the regression models, the EOA was implemented for predicting binding strength, while also highlighting performance metrics and selected features. The best classification and regression models were used on the TR dataset, which led to the creation of a small library of possible TR interactions with other proteins. Finally, MD simulation was performed on a selected PPI (TAS2R41 bitter receptor and the CHMP4A protein) among the top TR interactions to further explore their interaction behavior at an atomistic level. The flowchart of the proposed method is presented in Fig. 1.

### Binary PPI prediction results

Evaluation metrics were computed for BML and a multi-objective Pareto-based EOA. The performance of the binary classifier was evaluated using a 10-fold stratified cross-validation (CV) and an independent test set. For both methods, the CV and testing metrics of their respective best model are displayed (Table 1, Supplementary Fig. 1) by averaging the results of the individual runs (10 runs). For the BML, the model achieving the highest CV Accuracy (88.12%) was XGBoost (XGB) with a simple imputation method and was selected as the best model. For the EOA, the model with the highest CV Accuracy (88.22%) was trained using the RF classifier with 205 Trees, and the features selected by this model were: “MF_similarity,” “CC_similarity,” “Exists in MINT?,” “Exists in APID?,” “Exists in BIOGRID?,” “pfam_interaction,” “A %,” “I %,” “V %,” “S %,” “C %,” “W %,” “R %,” and “cys_reduced_dif” (see Supplementary Table 2 for the full analysis of features). While BML and EOA showcase similar metrics in the CV (except ROC-AUC, where EOA’s score is higher), EOA outperforms BML in the testing set in almost all the metrics.

**Table 1:** Performance of the binary classifier using benchmark machine-learning techniques (ML) and the multi-objective Pareto-based evolutionary optimization algorithm (EOA)

	Method	Accuracy	Specificity	Sensitivity	F1	F2	ROC-AUC
**CV**	BML	88.12 ± 0.03%	100.0 ± 0.06%	64.36 ± 0.06%	78.32 ± 0.04%	69.3 ± 0.05%	82.18 ± 0.0%
	EOA	88.16 ± 0.08%	98.79 ± 0.02%	66.89 ± 0.29%	78.95 ± 0.21%	71.23 ± 0.27%	91.5 ± 0.05%
**Test set**	BML	75.62%	98.63%	52.61%	68.34%	57.95%	75.62%
	**EOA (best)**	**77.73** ± **0.17%**	**97.38** ± **0.03%**	**58.14** ± **0.32%**	**72.34** ± **0.27%**	**63.09** ± **0.31%**	**78** ± **0.0%**
	EOA (ensemble)	77.20 ± 0.16%	98.17 ± 0.05%	56.29 ± 0.28	71.20% ±0.25	61.44 ± 0.27%	77 ± 0.0%
	STRINGDB	58.54%	99.77%	17.32%	29.47%	20.74%	58.55%
	D-SCRIPT*	66.31%	98.17%	6.61%	12.02%	8.06%	53.7%
	Topsy-Turvy*	67.97%,	87.89%	29.48%	38.57%	32.55%	60.94%
	EOA (best)*	**96.03** ± **0.17%**	**97.22** ± **0.33%**	**93.83** ± **0.13%**	**94.33** ± **0.22%**	**94.03** ± **0.33%**	**95.52** ± **0.10%**

Metrics are provided for cross-validation (CV) and testing sets. For the testing, metrics are also provided for the D-SCRIPT, Topsy-Turvy, and STRINGDB models. Asterisk (*) refers to the models that used the reduced version of the test dataset, along with the final number of test PPIs used and the ratio of positive/negative instances. “EOA (ensemble)” testing references to the ensemble testing method based on majority voting of the Pareto Front models, while EOA (best) references to the Pareto Front best model testing method. The metrics of the best-performing method are outlined in bold.

*Used Reduced Test Dataset: 34,362 PPIs (negative instances: 22,644, positive instances: 11,718).

We also performed a comparative evaluation of our methodology against state-of-the-art PPI prediction tools using the independent testing dataset. The tools included D-SCRIPT^48^, Topsy-Turvy^49^, and STRINGDB^50^. D-SCRIPT is a DL-based method designed to predict physical PPIs by leveraging sequence information and incorporating a structurally inspired architecture that enhances cross-species generalizability and interpretability through an estimated inter-protein contact map. Topsy-Turvy extends D-SCRIPT by integrating sequence-based inference with global network properties via transfer learning, enabling robust, genome-scale PPI predictions even in species lacking experimental PPI data. STRINGDB, in contrast, aggregates PPI predictions from a wide range of sources, including experimental data, computational predictions, and literature mining, to provide a comprehensive view of functional protein associations across numerous organisms. It is important to note that both D-SCRIPT and Topsy-Turvy were applied only to a reduced subset of the testing dataset. This limitation was due to missing sequence or ENSEMBL ID information required for embedding and mapping PPIs. Nonetheless, the reduced dataset was also evaluated using the best-performing EOA model, which achieved an even higher testing accuracy (96.03%) on this subset compared to its performance on the full test set.

A focused analysis on sensitivity across all models reveals a consistent challenge. While overall performance metrics such as accuracy and specificity remain high, sensitivity is markedly lower both in our models and in the benchmark tools. This issue is particularly pronounced in external tools like STRINGDB, D-SCRIPT, and Topsy-Turvy. The reduced sensitivity across methods can largely be attributed to the high rate of false positives (FPs) in experimental datasets, which introduces significant noise and makes it difficult to correctly identify true positive interactions. This reduced sensitivity highlights the challenge of identifying true positive interactions in noisy experimental datasets, underscoring the robustness of EOA despite these limitations. Despite these challenges, the proposed EOA method outperforms other baseline methods in both CV and test set evaluations, achieving the highest testing accuracy (77.73% on the full test set and 96.03% on the reduced-imbalanced one) (Table 1, Supplementary Fig. 1B).

### Regression results

The endpoint used for the affinity regression models training and testing was the Δ*G* values from the binding affinity Dataset as described in the “Methods” section “Binding affinity dataset.” Table 2 presents the average training, CV, and testing regression performance metrics for ten different runs of the proposed algorithm. The run that achieved the best correlation results based on the test set of 104 samples (Run 2- 0.1079 CV RMSE, 0.25 CV Correlation, 0.20 Ensemble Testing Correlation) was chosen as the best run. From this run, we used the ensemble of the Pareto Front models to perform affinity predictions on the positive instances of the TR dataset (predicted using the EOA best model). From this best run, we also provide information on the best-scoring model (0.9880 RMSE, 0.36 Correlation), which was trained using radial basis function (RBF) SVM with 140 Support Vectors. The features selected by this model can be found in Supplementary Table 12, while a detailed analysis of the features is presented in Supplementary Table 2. The statistically significant Spearman’s correlation of 0.22 in the CV, 0.19 in testing fall within the medium range, as values around and above 0.2 generally indicate moderate relationship between the predicted and actual values, suggesting that the fitted regression model is not able to accurately predict binding affinity of PPIs but can be used to rank them, as indicated by the RMSE metrics.

**Table 2:** Performance metrics for the regression analysis

	RMSE	RMSE (Δ*G*)	RAE	RRSE	Spearman correlation	Spearman *p* value
Training total	0.0951 ± 0.005	8.12 ± 0.42	86.03% ± 5.38%	83.82% ± 4.67%	0.81 ± 0.108	1.56e^−60^
CV total	0.1082 ± 0.0008	9.24 ± 0.07	97.78 ± 0.88%	98.72% ± 0.67%	0.22 ± 0.02	3.28e^−05^
Ensemble testing	0.1233 ± 0.0004	10.53 ± 0.03	99.69% ±0.72%	98.54% ± 0.41%	0.19 ± 0.01	0.05 ± 0.01

Metrics are provided for cross-validation (CV), training, and testing sets. RMSE values are provided for both the normalized Δ*G* values of the endpoint (0–1 range) and for absolute Δ*G* values (RMSE (Δ*G*)). Ensemble testing references to the ensemble testing method based on majority voting of the Pareto Front models.

*RMSE* root mean square error, *RAE* relative absolute error, *RRSE* root relative squared error.

### Taste receptor dataset analysis results

The end goal of this analysis was the use of the classification and regression models on a dataset that encapsulates the TRs interactome as discussed in the “Methods” section. First, these 816,518 interactions involving TRs were classified into interacting and non-interacting. This was performed using the classification model with the highest Accuracy (Best Model) from the Pareto Front of the run that achieved the best CV accuracy (Accuracy 88.22%). Using this method, 35,320 interactions were classified as positive (1), and the rest were classified as negative (0). Additionally, the prediction probabilities of the positive classes were also calculated. Secondly, for choosing the regression model(s) that will be used for calculating the binding strength of the positive TR interactions, we tested the ensemble of the Pareto Front models of each regression run on the regression test dataset (104 samples). The ensemble of the models of the run with the best testing correlation was chosen to evaluate the positive PPIs, predicting the binding strength score for each of them by majority voting. The TR interactions were ranked based on the mean of the predicted probabilities and the predicted binding strength scores. The library of the top 10 TR interactions is presented in Table 3, summarizing the top-ranked interactions overall, and providing an overview of the library’s format. The overall best 20 interactions, along with the best interactions of each taste’s receptors, are provided separately in Supplementary Tables 6–11. The best interactions were ranked according to the mean of their predicted affinity value and the classifier’s probability. These tables constitute a small library of possible TR interactions out of the vastness of the TR interactome, which needs further investigation by MD and experimental techniques to be validated.

**Table 3:** Library of the top 10 TR interactions overall

TR interaction (UID)	Gene1	Gene2	Probability score	Aff predictions (kJ/mol)	Mean Prob—Scaled Aff	iRefIndex check
P0C0E4-P59551	RAB40AL	TAS2R60	0.99	38.90	0.70	0
A1A580-Q7RTM1	KRTAP23-1	OTOP1	0.98	38.90	0.69	0
P37088-P51170	SCNN1A	SCNN1G	0.98	38.04	0.69	0
P05771-P41180	PRKCB	CASR	0.98	38.04	0.69	1
P59540-Q9NY47	TAS2R46	CACNA2D2	0.97	38.04	0.68	0
Q92556-Q9NYW3	ELMO1	TAS2R7	0.98	37.19	0.68	0
P49753-Q13255Β	ACOT2	GRM1	0.97	38.04	0.68	0
Q8WYK1-Q9NYW5	CNTNAP5	TAS2R4	0.97	38.04	0.68	0
P37088-Q9NRS4	SCNN1A	TMPRSS4	0.98	37.19	0.68	0
P21439-Q7RTM1	ABCB4	OTOP1	0.97	38.04	0.68	0

“TR interaction (UID)”: taste receptor interaction’s Uniprot IDs, Gene1/ Gene2: taste receptor interaction’s gene names, predicted classes: indication of the positive class prediction (1), “iRefIndex check”: 1 if the interaction has been recorded in iRefIndex, 0 if not, probability score: the classifier’s prediction probability score, “Aff predictions”: the binding strength (affinity) predictions of the regressor in absolute Δ*G* values, “Mean Prob—Scaled Aff”: the mean of classifier’s prediction probability score and the predicted binding strength in 0–1 scale.

**Fig. 2:**
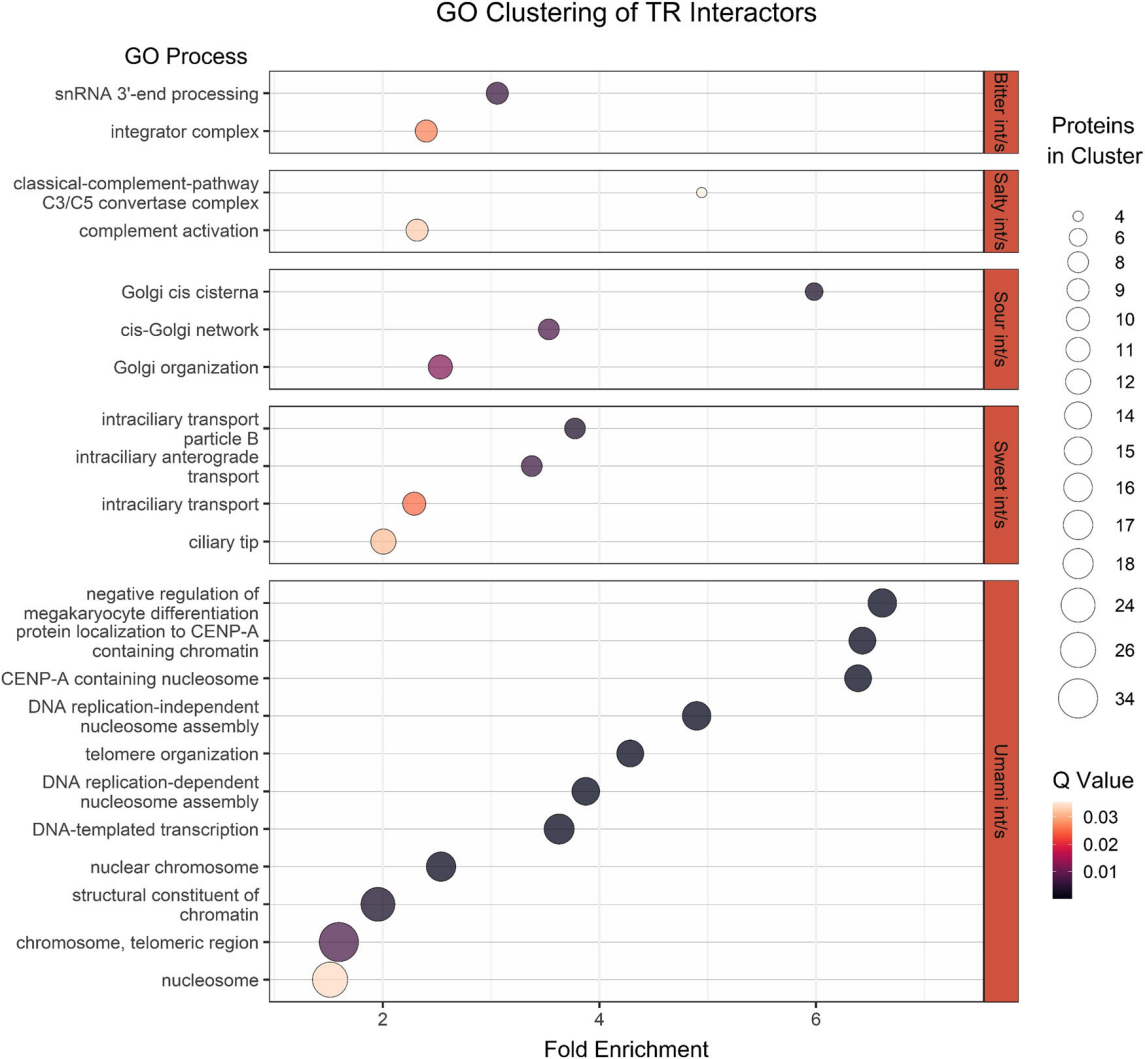
Enrichment analysis of the interactors of umami, sweet, salty, sour, and bitter receptors. The *y*-axis represents the GO term clusters that formed, grouped by each taste receptor’s interactors. The *x*-axis represents each cluster’s Fold Enrichment value, the color gradient represents its *q* value, and each dot’s size represents the number of proteins involved in the cluster.

Furthermore, functional enrichment analysis using GO terms was performed for all the non-TR proteins interacting with TRs (TR interactors) from each interacting pair (5 enrichment analyses for all the interactors of umami, sweet, sour, salty, and bitter receptors separately). The proteins studied in this analysis were all the positive PPI predictions from the TR dataset. The software used for the enrichment analysis was DAVID^51^. The classification stringency for DAVID was set to default Medium, and the cluster with the highest enrichment score and only the GO term clusters with *p*-adjusted value < 0.05 were used for the visualization. The results of the GO term enrichment analysis are presented in Fig. 2. The analysis revealed distinct functional enrichments for the interactors of each TR. Bitter interactors were enriched in GO terms associated with integrator complex formation and snRNA 3′-end processing. Sour interactors showed enrichment in GO terms related to Golgi network structure and organization. Sweet interactors were associated with GO terms linked to intraciliary transport mechanisms. Umami interactors exhibited enrichment in GO terms pertaining to chromatin and nucleosome organization. Finally, salty interactors were enriched in GO terms related to complement activation and the C3/C5 convertase complex; however, the *q* value for this cluster was relatively high, and the number of associated proteins was lower compared to the other taste interactors.

Additionally, five interaction networks were created using the best-scoring interactions involving (a) bitter, (b) sour, (c) sweet, (d) salty, and (e) umami receptors each time. Due to the high volume of interactions, a filter was applied in the positive interactions (35,320), and a threshold was applied to the classifier’s predicted probabilities (prob > 0.55) to focus on the top-ranked interactions. Thus, the final number of interactions involved in the visualized network was 159 (57 bitter, 28 salty, 6 sour, 5 sweet, 20 umami- TAS1R3 protein is involved in both sweet and umami networks). The Cytoscape^52^ software was used for the visualization of the network graphs. The results are presented in Supplementary Fig. 2. The full network of interactions involving all the best bitter receptors without filtering is presented in Supplementary Fig. 3.

**Fig. 3:**
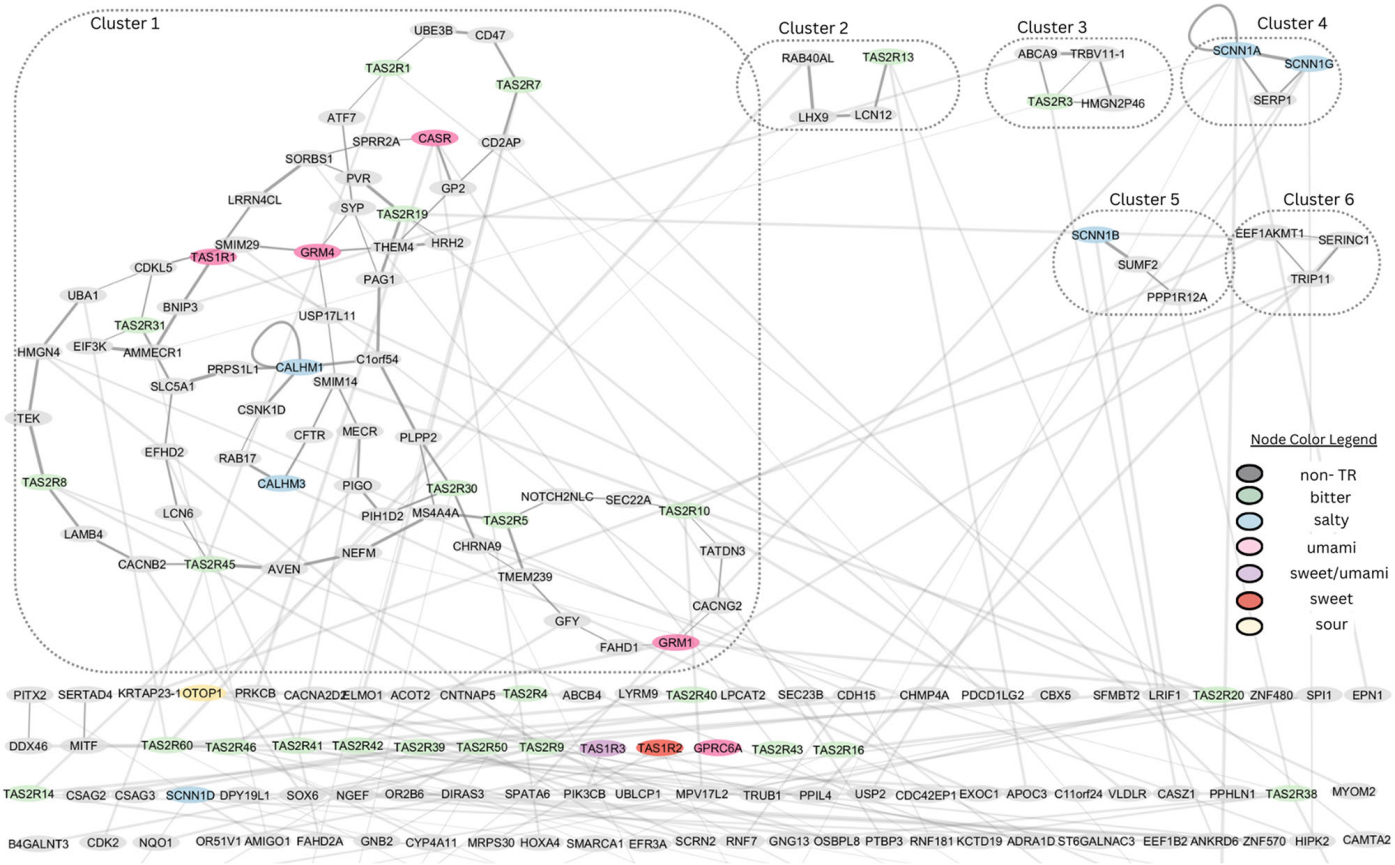
Molecular Complex Detection (MCODE) clustering of the interaction network of the top-scoring interactions. Node color is indicative of the TR type. More specifically, bitter TRs are represented in green, salty in light blue, umami in pink, sweet in red, sour in light yellow, and non-TRs in gray. The sweet/umami (purple) in the legend refers to the TAS1R3, which is the monomer forming both the sweet and the umami receptors. Edge width is analogous to the mean of the classifier’s probability score and the predicted binding strength of the interaction. Transparency is higher for the inter-cluster edges, while intra-cluster edges are non-transparent. Dotted lines enclose the different cluster nodes (Clusters 1–6). Edges were added to the network based on the predicted interactions using the best-performing PPI ML model.

Furthermore, the Molecular Complex Detection (MCODE) clustering algorithm^53^ was used on the full interaction network of the 159 TR interactions (prob > 0.55) and the corresponding positive interactions of their interacting pairs (187 additional +PPIs involving only the non-TR interactors of these 159 PPIs). In this graph, the nodes were colored according to their TR type (bitter, sweet, salty, sour, umami, sweet/umami, non-TR), and the end goal was to assess the clustering of TR types. The parameters for the MCODE algorithm were set on degree cutoff 1, node score cutoff 0.5, k-Core 2, and max depth 100. The results of the clustering are presented in Fig. 3.

Six distinct clusters (Clusters 1–6) were identified through the analysis of the full interaction network. Cluster 1 appeared to be the most extensive, both in terms of interaction diversity and the number of connections, encompassing interactions among bitter, salty, and umami TRs. Clusters 2 and 3 consisted of four nodes each, representing interactions of the bitter receptors TAS2R13 and TAS2R3, respectively, with non-TR proteins. Clusters 4–6 were of less biological significance and are composed of three nodes, with clusters 4 and 5 involving interactions exclusively among salty receptors.

### Molecular dynamics results

**Fig. 4:**
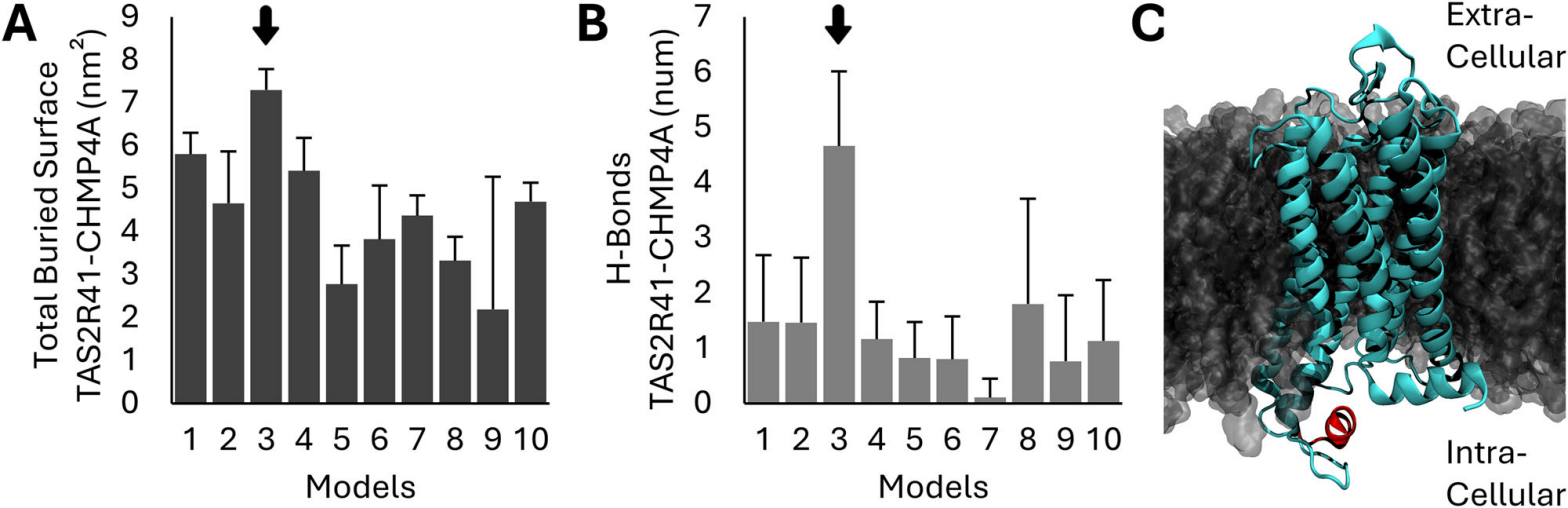
Total buried surface and H-Bonds analyses of the interaction between TAS2R41 bitter receptor and CHMP4A protein. **A** Total buried surface (TBS) analysis between TAS2R41 bitter receptor and CHMP4A protein of the 10 models. The highest total buried surface value between the 10 models is highlighted by the black arrow. **B** H-Bonds analysis between TAS2R41 bitter receptor and CHMP4A protein of the 10 models. The highest H-Bonds value between the 10 models is highlighted by the black arrow. **C** Qualitative representation of the third model inside the POPC membrane. In green it is shown the TAS2R41 bitter receptor, while in red it is shown the CHMP4A protein.

MD was adopted as the main tool to investigate the PPI pair selected following the criteria listed in the “Methods” section “Molecular dynamics.” The TAS2R41-CHMP4A protein pair was selected for MD simulation based on criteria ensuring structural availability, computational feasibility, and sufficient binding site information (see detailed rationale in the “Methods” section “Molecular dynamics”). TAS2R41 (UniProt ID: P59536) and CHMP4A (UniProt ID: Q9BY43) met all requirements, including having PDB structures and being under 350 amino acids (Supplementary Table 6). The crucial step to elucidate PPIs involves the identification of optimal binding poses. A common approach to discerning highly interacting poses from weakly interacting ones is through the analysis of total buried surface (TBS) area and hydrogen bond formation^54^. Therefore, TBS and H-Bonds analyses have been performed between the TAS2R41 bitter receptor and CHMP4A protein in all 10 docked model systems (Fig. 4). Notably, Model 3 exhibited the highest TBS value, with a mean value of 7.30 ± 0.49 nm^2^ (Fig. 4A). Similarly, Model 3 displayed the highest number of hydrogen bonds formed between the two proteins, with a mean value of 4.66 ± 1.35 (Fig. 4B). In addition, a qualitative representation of Model 3 is shown in Fig. 4C. The findings from these initial analyses indicate that the interaction between the TAS2R41 bitter receptor and CHMP4A protein, starting from the conformation of the docked Model 3 resulted strong in both TBS and hydrogen bonds. Furthermore, the average TBS and H-Bonds of Model 3 are notably higher, even when considering the standard deviation of all other models. This reaffirms the presence of a highly valuable conformation that needs further in-depth analysis.

**Fig. 5:**
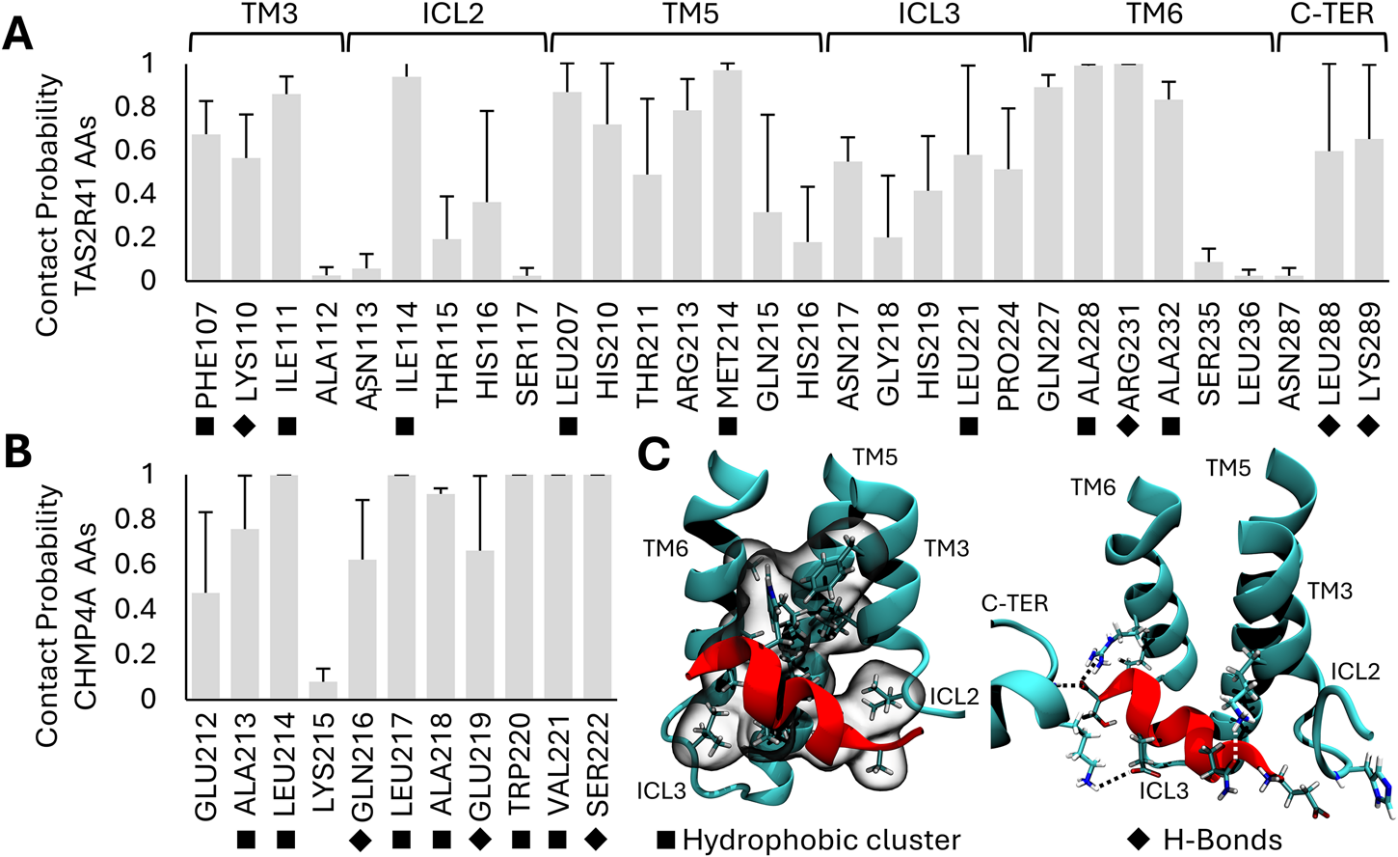
Contact probability analysis of TAS2R41 bitter receptor and CHMP4A protein. **A** Contact Probability (CP) of TAS2R41 bitter receptor divided per residue. Only residues with CP > 0.02 are represented. In addition, a schematic representation of the bitter taste receptor structural moieties, including the intracellular loops (ICLs), the transmembrane (TM) helices, and the C-terminal section (C-TER), is highlighted. **B** CP of CHMP4A protein divided per residue. **C** Qualitative depiction of the hydrophobic cluster (on the left) and H-Bonds (on the right) formed between the TAS2R41-CHMP4A interacting amino acids. Moreover, it is also shown the main hydrophobic amino acids involved in the hydrophobic cluster formation with black square marks. Instead, the amino acids involved in the H-Bonds formation are shown with black diamond marks.

To further characterize the interactions between TAS2R41 and CHMP4A in the Model 3 system, we performed contact probability (CP) analysis (Fig. 5). This analysis provides further insights into the dynamic behavior and stability of the protein–protein complex through the investigation of the most important amino acids responsible for the binding process. Specifically, the CP analysis was performed among each residue of the two proteins with a cutoff distance of 0.28 nm, as previously done in literature^55^. The results revealed several key amino acids of TAS2R41 strongly implicated in the binding process (CP > 80%), including ILE111, ILE114, LEU207, MET214, GLN227, ALA228, ARG231, and ALA232 residues (Fig. 5A). Furthermore, additional amino acids of TAS2R41 play a crucial role in the binding process (50% < CP < 80%), encompassing PHE107, LYS110, HIS210, ARG213, ASN217, LEU221, LEU288, and LYS289. Remarkably, among all the most relevant residues of TAS2R41 in the interaction mechanism with CHMP4A, there are 4 positively charged, 3 polar, and 9 hydrophobic amino acids. In a similar way, several amino acids of CHMP4A were identified as crucial for the binding process (CP > 80%), including LEU214, LEU217, ALA218, TRP220, VAL221, and SER222 (Fig. 5B). In addition to them, GLU212, ALA213, GLN216, and GLU219 also play a crucial role in the interaction mechanism (50% < CP < 80%). Notably, among all the most relevant residues of CHMP4A in the interaction mechanism with TAS2R41, there are 2 negatively charged, 2 polar, and 6 hydrophobic amino acids.

Noteworthy, our findings underscore the critical importance of hydrophobic interactions, given the prevalent abundance of hydrophobic amino acids within the CP profile in both proteins. Additionally, the CP analysis also emphasizes the importance of the electrostatic interactions, given the prevalence of positively charged amino acids in TAS2R41 and negatively charged amino acids in CHMP4A. Within this context, the qualitative depiction of the hydrophobic cluster and hydrogen bonding formed between TAS2R41 and CHMP4A is illustrated in Fig. 5C, providing visual insights into the structural basis of their interaction. The CP analysis complements the previously conducted TBS and hydrogen bond analyses, enhancing our comprehension of the molecular interactions governing the TAS2R41-CHMP4A complex.

**Fig. 6:**
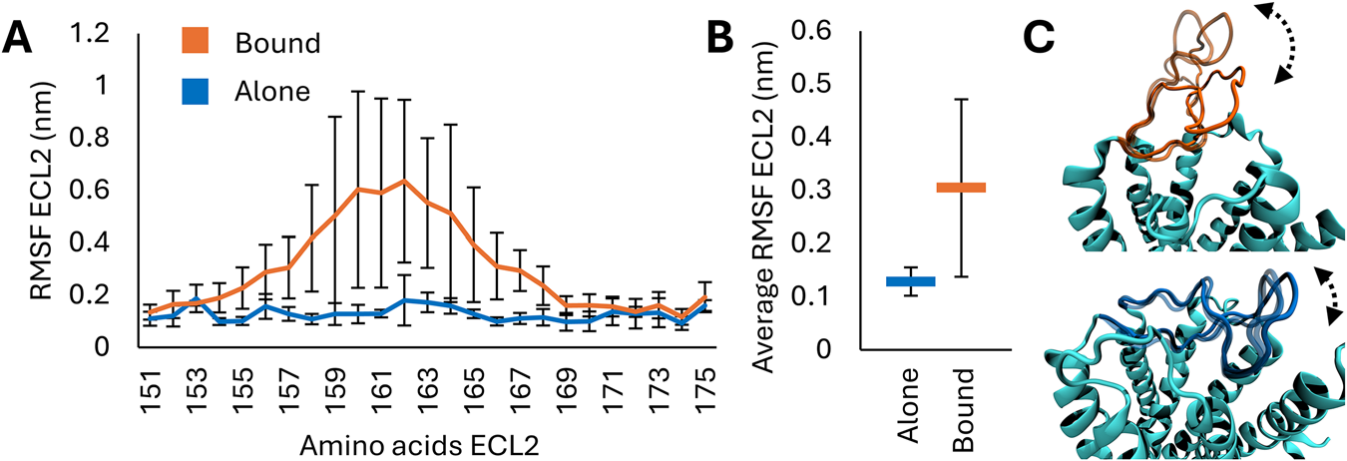
TAS2R41 root mean square fluctuation analysis. **A** Root Mean Square Fluctuation (RMSF) of the TAS2R41 external loop 2 (ECL2) divided per residue. In orange is shown the TAS2R41 bitter receptor in complexation with CHMP4A protein (Bound), while in blue is shown the standalone TAS2R41 bitter receptor (Alone). **B** Average of the RMSF values of all the ECL2’s residues. **C** Qualitative depiction of the ECL2 different ECL2 fluctuations between the complexed (top figure) and the standalone (bottom figure) TAS2R41.

Subsequently, we explored the potential impact of the close interaction between CHMP4A and TAS2R41 on the overall dynamic behavior of the latter. Visual inspection using Visual Molecular Dynamics (VMD)^56^ revealed increased fluctuation in the extracellular loop 2 (ECL2) section of TAS2R41. To quantify this movement, root mean square fluctuation (RMSF) analysis of the ECL2 section was performed for both the standalone TAS2R41 and the complex system (Model 3) (Fig. 6). Interestingly, the analysis showed a notable difference in the RMSF values, indicating that TAS2R41 in complexation with CHMP4A exhibited markedly higher RMSF values compared to the standalone TAS2R41 (Fig. 6A). More in detail, the greater difference in both average RMSF and standard deviation is localized in the ECL2 section comprising residues 154–169. To further highlight these differences, the average RMSF and standard deviation values of all the ECL2 residues were calculated for both cases. The results revealed that TAS2R41 in complexation with CHMP4A had an ECL2 average RMSF value of 0.31 ± 0.17 nm, whereas the standalone TAS2R41 exhibited an ECL2 average RMSF value of 0.13 ± 0.03 nm (Fig. 6B). It is worth mentioning that the average RMSF value of the TAS2R41 in complexation with CHMP4A exhibits nearly 2.5 times more fluctuations in comparison to the standalone TAS2R41. In this context, a qualitative depiction of the ECL2 different fluctuations between the standalone and the complexed TAS2R41 is shown in Fig. 6C. Given that the ECL2 plays a crucial role in occluding the binding pocket for bitter tastants, the elevated fluctuation in this region may facilitate greater accessibility to ligand binding, consequently leading to enhanced receptor activation.

## Discussion

The central focus of this study was to design a binary classifier and a regression model for the identification of PPIs and the quantification of their interaction strength using a diverse range of sequential, structural, homology, gene expression, and additional protein-specific information. The primary objective of our study was to identify novel interactions among TRs with other proteins and elucidate their potential roles in taste molecular pathways and functions. To this end, a feature calculation workflow was employed for constructing the appropriate training and testing datasets for the classification and regression models, while for the latter, additional endpoints were constructed using experimental binding affinities. Since we aimed to apply the overall method to explore the full human proteome, we did not include as inputs structural features that come from docking or other protein structure analysis that is either not available for most proteins or is not feasible to calculate for the overall dataset. Two different ML methodologies were employed for the model construction: a methodology based on the optimization of benchmark ML algorithms (BML method) and a multi-objective Pareto-based EOA methodology. The trained models can be applied to identify potential PPIs and quantify their strength, requiring minimum computational resources and thus making the overall solution suitable for screening novel interactions that could be then analyzed with more time-consuming molecular modeling or experimental techniques.

The proposed EOA method clearly significantly outperformed BML in classification performance, except for specificity, which was similar in the two metrics, while also ending up using fewer features. Based on these results, it was decided that the best method for testing the TR interactome dataset was EOA. Choosing between the two EOA testing approaches, it was decided that the best model approach was slightly more efficient than the ensemble one, thus concluding that the highest accuracy model (88.85% accuracy) of the EOA should be used for the testing.

It is noteworthy that sensitivity was considerably reduced compared to the rest of the metrics for all tested models. This is partially explained by the high FP rates contained in PPI databases containing experimental data. It is estimated that around half of the data generated by high-throughput methods may contain FP instances^57,58^, a phenomenon also occurring in Y2H filtered datasets^17^. Our training set contains only PPIs that were verified by the two most prominent experimental techniques, namely the Y2H and the Tandem Affinity Purification (TAP). Relying solely on these two methods inevitably restricts the positive dataset available for training our models. However, this limitation aligns with our primary objective of elucidating new molecular pathways of TRs with a high degree of confidence in the predicted PPIs, even if it means missing potential interactions. Moreover, extending the gold-standard set of positive experimentally verified PPIs can improve the accuracy of the PPI prediction models, allowing to overcome the class imbalance issues of the dataset. In addition, the classifier’s test prediction metrics significantly surpassed D-SCRIPT’s, Topsy-Turvy’s, and STRINGDB’s predictions on the reduced version of our test dataset, with all these models presenting very poor sensitivity (6.61%, 29.48%, and 17.32%, respectively). These methods were not able to perform predictions for the full test set, highlighting the need for community-standard benchmark datasets for comparative testing of PPI tools, a fact that was pointed out by studies on PPI datasets^59,60^. For this reason, we opted for comparatively testing the EOA in the reduced fraction of the test dataset for which D-SCRIPT and Topsy-Turvy were able to perform predictions. In this test set, version EOA provided much better classification metrics, and the performance was considerably higher than before across all metrics. This serves as an indication of the classifier’s predictive abilities in comparison to recent and popular PPI-DL tools and databases, against a comprehensive PPI test dataset. Our secondary objective was to curate a small database (Supplementary Tables 6–11) of possible PPIs for TRs that will undergo further investigation through MD simulations in future studies. Moving forward, to acquire enough samples for the testing set, this criterion was relaxed, allowing the inclusion of PPIs verified by multiple experimental techniques. Therefore, the testing set is expected to include the highest number of FPs, and thus, the reduced sensitivity of the models can be caused by a lack of overfitting. After applying the classification model to the 816,518 UniProt pairs of the TR interactome, 35,320 pairs were classified as positive (interacting).

The interactors of each taste’s receptors were enriched using GO terms and the DAVID software as shown in Fig. 2. The GO terms with the highest Fold enrichment value and the highest *Q* value are the following and are analyzed through relevant literature, drawing hypotheses on the potential role of specific TR interactors in the pathways of taste perception. The bitter interactors were enriched with GO terms involved in the integrator complex and the snRNA 3′-end processing. As is known, bitter receptors are GPCRs, and a main interactor of them is the Gβγ subunit, which is thought to interact with RNA polymerase II^61^, a key target of the integrator complex. This suggests that the bitter interactors may be involved with the function of the integrator complex. In turn, the integrator complex, through mRNA regulation, is involved in many TR mechanisms, such as in the production of ghrelin mRNA in salivary glands^62^. Ghrelin is an appetite-regulating hormone, and this may indicate a possible regulating mechanism of appetite by bitter receptors. The sour interactors were enriched with GO terms involved in the Golgi network’s structure and organization. OTOP1, the sour receptor, is an ion channel, and as such is subjected to post-translational modifications in the cisternae of the Golgi apparatus^63^. This explains the phenomenon of sour interactors being involved in the Golgi network organization. Furthermore, the sweet interactors were enriched with GO terms involved in intraciliary transport mechanisms. The intraciliary/intraflagellar transport (IFT) mechanism, and specifically the cilium length and function, are thought to be involved in odor detection^64^. Thus, the interactors of the sweet TRs may also trigger the olfactory receptors through the IFT mechanism, appearing in this way enriched in the corresponding GO terms. It is well-characterized that odor detection influences taste perception and its mechanisms through the neural pathways, and this finding may provide new insights into the study of this phenomenon^65–67^. The umami interactors were enriched with GO terms involved generally in chromatin/nucleosome organization. Previous literature pointed out that elevated levels of GPCRs (such as the umami receptors) trigger nuclear actin-dependent alterations in chromatin organization^68^. Therefore, the interactors of GPCRs may be indirectly involved with chromatin organization, explaining in this way the analogous GO term enrichment. Finally, the salty interactors were enriched with terms involved in the complement activation and the C3/C5 convertase complex, but it is worth noting that the *q* value of the clusters is relatively high, and the proteins involved in them are lower compared to the rest of the tastes’ interactors. GO enrichment results should be interpreted with caution, and can only be used to draw new biological hypotheses on the role of selected TR interactors and have to be experimentally verified by laboratory methods in order to confirm their biological significance.

Concerning the regression results, the reported RMSE values correspond to 8.12, 9.24, and 10.53 absolute Δ*G* values when normalization was inversed, meaning that the predicted values have an average deviation of 10.53 from the real binding affinity values. These results may not suggest a strong predictive ability from the regressor, but they demonstrate its ability to separate the low-affinity from the high-affinity PPIs, outperforming other known MD methods, such as MM-GBSA, thus providing an extra measure of ranking and identifying the most probable PPIs. The predicted Δ*G* values are not to be considered as accurate approximations of experimental affinity, and such interpretation is discouraged, but the RMSE deviation can be quantitative evidence of the ranking ability of the regressor.

Using a combination of the classifier’s probability score and the regressor’s predicted affinity, the 35,320 interacting pairs were further narrowed down to 159 PPIs that were used in the creation of an interaction network. In theory, such networks can be reorganized to reflect information about the tissue-specificity and relevant cellular compartments of its protein. However, such information is scarce and available only for a limited subset of the proteins, making such an endeavor infeasible with the current knowledge. The clustering analysis of the full interaction network identified six distinct clusters (Clusters 1–6). Cluster 1 was the most extensive, both in terms of interaction diversity and the number of connections, encompassing interactions among bitter, salty, and umami TRs. Clusters 2 and 3 consisted of four nodes each, representing interactions of the bitter receptors TAS2R13 and TAS2R3, respectively, with non-TR proteins. Clusters 4–6 were smaller, three-node clusters of lesser biological significance, with Clusters 4 and 5 involving interactions exclusively among salty receptors. Notably, the findings from Cluster 1 (salty-bitter-umami interactions) align with existing evidence highlighting the interplay between bitter, sour, and salty taste modalities^69^. Salty taste, for example, is shown to affect the bitterness of food through inhibition, while both sour and salty tastes interact to enhance their effect on low concentrations^70^. This interplay may provide useful information about the interaction networks of the TRs and demonstrate new mechanisms of parallel taste activation or inhibition. Furthermore, enrichment analysis for the Cluster 1 genes using the CORUM v5.0 database^71^ of human complexes showcased the involvement of GRM1 and calcium-sensing receptor (CASR) in the same complex as part of the G protein-coupled receptor signaling pathway, crucial for taste perception. The glutamate receptor family in humans contains 15 members, among which are eight metabotropic glutamate receptors (GRM1, GRM2, GRM3, GRM4, GRM5, GRM6, GRM7, and GRM8) and a single CASR^72^. Metabotropic glutamate receptor subtypes 1 and 4 (mGluR1, mGluR4) as well as CASR have been proposed as receptors of the umami taste in humans, and the G protein-coupled receptor signaling pathway is crucial for taste perception in general^6^. This provides further explanation of the findings from Cluster 1, involving the GRM1 and CASR genes. The interactions of TRs in the aforementioned clusters are to be verified and analyzed by other more advanced techniques, such as MD or experimental analyses in order for meaningful biological validation. This analysis highlights the most probable interaction clusters based on network techniques and available bibliography and helps researchers elucidate the mechanisms of taste perception. Overall, the GO term clustering and network analyses are alternative, useful techniques to help narrow down the multitude of identified TR interactions and help identify potential interactions of interest.

To validate and gain insights into the conformational dynamics of the predictions of our model, we performed MD simulations on the TAS2R41-CHMP4A PPI (Supplementary Table 6). In detail, it is used in the section spanning from GLU212 to SER222 of the CHMP4A protein. This selection was motivated by the presence of two experimental PDB structures where this moiety is known to participate in interactions with other proteins^73,74^. By targeting this region, we aimed to explore the potential interaction interface and dynamics between TAS2R41 and CHMP4A in a biologically relevant context. The TAS2R41 bitter receptor and CHMP4A protein were initially docked, and subsequent MD simulations were performed on the first 10 docked models. Our analysis revealed that Model 3 exhibited the highest TBS and H-Bonds between the two proteins compared to all other systems (Fig. 4). This observation suggests that Model 3 represents a particularly stable conformation of the TAS2R41-CHMP4A complex, highlighting its potential biological relevance. It is interesting to note that the CHMP4A protein is complexed to the TAS2R41 bitter receptor in the transmembrane (TM) 5, internal loop 3 (ICL3), and TM6 regions. Interestingly, these specific regions are involved in the G protein interaction, playing a crucial role in the GPCR activation^75,76^. In literature, it has been highlighted that the ICL3 plays a critical role in TAS2R4 bitter TR activation^77^. Moreover, it was previously shown in other GPCRs that the proper conformation of ICL3 is important for receptor folding and function^78–81^. These observations are particularly relevant considering the region of interaction between TAS2R41 and CHMP4A, which encompasses the TM5-ICL3-TM6 region.

To fully elucidate the interactions between CHMP4A and the TAS2R41’s TM5-ICL3-TM6 regions, it has been performed the CP analysis (Fig. 5). Interestingly, CHMP4A can coordinate not only with the TAS2R41’s TM5-ICL3-TM6 moieties, but also with the TM3-ICL2 and the C-TER moieties (Fig. 5A). Furthermore, several amino acids of CHMP4A were identified as pivotal players in the complexation process, including LEU214, LEU217, ALA218, TRP220, VAL221, and SER222 (Fig. 5B). It is worth noting that LEU214, LEU217, and TRP220 amino acids play a crucial role in the complexation of CHMP4A with other proteins^73,74^. Indeed, they are fundamental for the formation of the hydrophobic cluster with TAS2R41’s TM5-ICL3-TM6 sections. Although the hydrophobic interactions are of primary importance for the binding process, the electrostatic stabilization between LYS110, ARG231, and LYS289 of TAS2R41 and GLN216, 219GLU, and SER222 of CHMP4A also plays a crucial role in the complexation process (Fig. 5C). Furthermore, the RMSF analysis of the ECL2 revealed higher fluctuation in the complexed model compared to the standalone one (Fig. 6), suggesting potential conformational changes induced by the interaction with CHMP4A. The increased fluctuation of the ECL2 in the TAS2R41-CHMP4A complex can lead to an increased propensity of bitter tastants to enter the TAS2R41 binding pocket, promoting receptor activation^75,82^. In this context, the TM5-ICL3-TM6 region targeted by CHMP4A can also hypothetically trigger a constitutively active state of TAS2R41. This observation aligns with previous literature indicating the critical role of ICL3 in the activation of GPCRs^77,83^.

The hypothetical activation of TAS2R41 by CHMP4A could open new avenues of research, as the CHMP4 protein family is heavily involved in the endosomal sorting complex required for transport (ESCRT) machinery^74,84^. The ESCRT system plays a vital role in repairing damaged plasma membranes, removing membrane vesicles from cells, and maintaining cell survival^85^. From another perspective, TAS2R41 is a highly selective bitter receptor^86,87^, and recently it has been discovered that it is hyper-specialized in chloramphenicol recognition^88^. In this sense, studying the TAS2R41 bitter receptor in conjunction with other proteins may also uncover novel interactions that could contribute to our understanding of antibiotics, such as chloramphenicol, and their secondary mechanisms of action. In conclusion, our study not only validated one of the top PPIs predicted by our model through MD investigation but also provided insights into the interaction mechanisms between TAS2R41 and CHMP4A. Furthermore, an additional MD study of the TAS2R41-EFHD2 interaction is provided in Supplementary Fig. 4 in the supplementary file.

However, further studies are needed to elucidate the functional significance of other predicted PPIs and their implications in taste perception and molecular pathways. In addition, experimental validation remains crucial to confirm the predicted interactions. Exploring more deeply how some of the pathway patterns that were studied are already known for other biological functions and involved in specific pathologies besides their role in taste perception, facilitating targeted nutraceutical design and drug discovery. Computational tools like, HDOCK^89^, HADDOCK^90^, free energy perturbation^91^, thermodynamic integration^92^, umbrella sampling^93^, fast pulling of ligand scheme^94^, and MM-GBSA^95^ can be used to obtain the proteins’ docking poses and their estimated binding strength^96–98^ as another method to confirm ML-predicted PPIs. However, these methods are not an optimal solution for calculating Δ*G* values, as they come with numerous pitfalls, including reliance on extensive MD searches, high computational cost, and inherent methodological limitations that can compromise accuracy and applicability. VirtuousPocketome tool^99^, a more descriptive tool that has been recently developed, provides accurate structural motif description of complexes using large database screening. These in-silico tools can be used as a launching point to gain insights into the morphological aspects of the PPI modes and their binding strength. However, experimental techniques are still required to properly validate the predicted PPIs, such as SPR^35^, ITC^36^, FRET^37^, BLI^38^, and Dynamic Light Scattering^100^.

In this study, we employed ML methods coupled with molecular modeling techniques to resolve and study the TR interactome. To achieve this objective, we introduced and benchmarked an EOA model for predicting TR-associated PPIs and estimating their binding affinity. In addition, this work also led to the curation of a small database of potential TR interactions with other proteins, which will require further study to be fully elucidated. As an example of the applicability of the TR interactome, we were able to validate and investigate novel TR interactions of TAS2R41 bitter receptor using MD. This discovery underscores the importance of ML techniques in synergy with MD tools in comprehending PPIs and their relevance to TR-associated pathways. By continuing to explore the functional significance of predicted PPIs and their implications in taste perception and molecular pathways, we aim to contribute to a deeper understanding of TR biology and its potential applications in various fields, including drug discovery and personalized nutrition.

## Methods

The flowchart of the proposed method is presented in Fig. 1, which follows a detailed description of the process from building the training and testing datasets for the classifier and regression models to the model creation and evaluation, and eventually to the molecular modeling analysis of the results.

### PPI dataset building

The launching pad for the application of ML algorithms was the creation of a well-curated and abundant dataset. In the case of PPI prediction, the first step in creating the dataset is to collect candidate positive and negative PPIs from existing databases. For instance, one of these databases used in the creation of the PPI dataset is iRefIndex^101^, a consolidated, non-redundant protein interaction database containing 3,654,531 PPIs, of which 1,647,374 are unique. Furthermore, iRefIndex consolidates interactions from a plethora of databases such as BIND^102^, BioGRID^103^, DIP^104^, HPRD^105^, IntAct^106^, MINT^107^, MPact^108^, MPPI^109^, and OPHID^110^. These state-of-the-art databases are used extensively by multiple ML-PPI tools for training and validation purposes, remaining the first choice for modern applications, and the main databases for reference in PPI studies^111,112^. Furthermore, iRefIndex maps proteins and PPIs to redundant groups using SEGUID-based keys, while filtering out malformed and deprecated identifiers, incorrectly assigned taxonomy identifiers, and ambiguous mappings. This whole unified and consolidated approach by iRefIndex was the reason for its selection as the main database for PPI mining, facilitating the study of the majority of experimentally verified interactions from golden-standard public databases. Negative protein interactions were obtained by the human negative PPIs dataset created by Trabuco, Betts, and Russell^113^ (Russell’s Negative Dataset for abbreviation), which contains 894,213 binary interactions (in the form of Uniprot IDs) that were derived from Y2H experiments and then validated by other experimental data. Even though experimental PPI data were used in both positive and negative cases, the persistence of FP and false negative (FN) cases remains an issue in database selection^114^. However, Trabuco et al., in previous research, have extensively curated the unobserved interactions by selecting those that were actually tested in the two-hybrid screens via viability analysis^113^. It is important to note the ongoing debates about the accuracy of negative PPI datasets, as the persistence of FN rates in them and the heuristic random selection of PPIs as non-interacting have prevented the creation of gold-standard data. For this reason, in our mining approach, we proposed the use of Russell’s Negative Dataset as a benchmark dataset due to its experimentally verified approach and efforts of FN rate reduction^115,116^. However, still the absence of a golden-standard PPI dataset persists as a limitation of all computational PPI predictors.

The training dataset consists of positive PPIs derived from iRefIndex after filtering, and twice the number of negative PPIs derived from Russell’s Dataset. This ratio of negative to positive samples is chosen to reflect biological systems, where the number of non-interacting protein pairs significantly exceeds that of interacting pairs, leading to a natural imbalance, which is to be accounted for in PPI studies^117–119^. Filtering of the iRefIndex was performed by retaining only PPIs where both interacting proteins are identified by their UniProt ID. Furthermore, only the unique PPIs were kept, using the Checksum Interaction method described in the iRefIndex documentation. Rows with identical RIGIDs (redundant interaction group identifiers) all describe interactions between the same set of proteins, so the PPIs were grouped by keeping unique RIGIDs. The iRefIndex’s “Checksum_Interaction” column may be used to identify other rows (interaction records) in this file that describe interactions between the same set of proteins from the same taxon ID. Further filtering was performed to keep only PPIs that have been experimentally verified with state-of-the-art methods of interaction detection, such as Y2H and TAP. Ultimately, only binary PPIs involving Homo Sapiens as the host organism were selected for further analysis. After applying these filtering criteria, a total of 1678 positive PPI entries remained. Then, the training dataset was completed by adding double the number of negative PPIs (3356). With these additions, the full training dataset consists of 5,034 PPIs. Additional information regarding the filtering can be found in the Supplementary Table 1. With these additions, the full training dataset consists of 5034 PPIs. Additional information regarding the filtering can be found in the Supplementary Table 1.

The same filters that were used in the curation of the positive training dataset were also applied to obtain the positive PPIs in the testing dataset. The main difference was that all the PPI detection methods recorded in iRefIndex were included, except for Y2H and TAP, which were the filters used for the creation of the positive training dataset. We thus reached 25,438 positive PPIs in the test dataset. For obtaining the negative PPIs, the following parameters were considered: first, the original Russel’s Negative Dataset was filtered to exclude the negative interactions considered in the training dataset. The original dataset was therefore reduced from 894,213 PPIs to 890,857 PPIs (exactly the 3356 PPIs used in the training dataset). Secondly, 25,438 interactions were randomly selected from the reduced Russel’s dataset to match the number of positive ones. Then, the dataset was checked for potential duplicates in the positive test or training datasets. Indeed, 6 duplicates were found and removed, reducing the negative PPIs to a total of 25,432. The use of highly curated PPIs in the training dataset ensured that the model learns from the most reliable and high-confidence interactions while relaxing these constraints in the testing dataset to evaluate generalizability. Restricting the training data to interactions detected by Y2H and TAP-MS minimizes noise and ensures that the model learns biologically meaningful patterns rather than potential FP interactions. In contrast, the test dataset includes PPIs detected by all available experimental methods except Y2H and TAP. This design helps test for overfitting to specific experimental methods, and adequate test performances ensure that the model can generalize beyond the data it was trained on. Additionally, using a large and diverse test set, even at the cost of potential training performance, provides a more robust and realistic evaluation of the model. This balance between a highly curated training dataset and a broader, more inclusive testing dataset allows us to optimize learning while ensuring meaningful evaluation of our model’s predictive capabilities in potential real-world PPI network scenarios.

Finally, we performed a similarity check on the PPI pairs of the testing set to identify any similar pairs with the training dataset. We used a BLASTP *E*-value < 0.05 threshold, and if both proteins in any testing pair were below that threshold when compared with both proteins in any other pair in the training dataset, they were excluded. *E*-values below this threshold indicate significant sequence similarity and homology^120^, and by the use of this threshold, we aimed to ensure that our model’s performance in the testing phase was not artificially inflated due to highly homologous protein pairs being present in both training and testing sets. Thus, this threshold was preferred over a stricter threshold (e.g., 0.001) to remove all potentially similar protein pairs. Indeed, 394 PPIs were excluded from the dataset due to similarity reasons. The overall testing dataset consists of 50,476 PPIs (25,271+ and 25,195−).

### Feature calculation

The features calculated on the previously described dataset were in total of 61. In the general case, as described by literature, PPI computational predictors utilize features such as protein sequence, protein structure, domain-domain interactions, gene expression information, AAC, domain/motif composition, or hydrophobicity profiles of input protein sequences, interface properties of protein 3D structures, gene neighboring or phylogenetic relationship^23,111,112^. The feature set used in this study takes into account all this available information, by selecting and curating the most interaction-informative features without redundancies. Specifically, these 61 features combine functional similarity, orthologous interactions in other organisms, PPI data mined from other databases, sequential, co-expression, and structural information of the proteins of each potentially interacting pair, expanding a previous PPI prediction dataset with additional features^121^. The features used for our dataset include three functional similarity features highlighting the similarity of the proteins in the pair based on the similarity of their respective GO terms. The GO terms are filtered, keeping only those that are indicative of a biological process, a molecular function, and a cellular component. Four features describe the existence of a homologous interacting pair in other organisms, specifically in *M. musculus*, *D. melanogaster*, *S. cerevisiae*, and *E. coli*. Four more features indicate if a PPI is recorded in other databases as well, while a “Sequence Similarity” feature contains the *E*-value of the two protein sequences. The “pfam interaction” feature indicates the presence of known domain interactions between the PPI pair. If two proteins share at least one interacting pair of Pfam IDs, the pair is marked as having a known domain interaction. Subcellular co-localization of the two proteins in the pair is indicated by one feature. Then, 19 features are calculated to measure the similarity of the two proteins in the PPI pair in terms of their Gene and RNA Expression among 17 gene expression datasets. The exact gene expression datasets are: GDS531, GDS534, GDS596, GDS651, GDS806, GDS807, GDS843, GDS987, GDS1085, GDS2855, GDS1402, GDS181, GDS1088, GDS841, GDS3257, GSE227375, and GSE228702, while the RNA expression datasets are GSE227375 and GSE228702, from NCBI Gene Expression Omnibus. For each dataset, each protein is matched with its corresponding expression profile, and then Spearman correlation indexes are calculated between the two proteins of each PPI pair. 30 features represent the absolute difference in numerical protein features between the proteins of each PPI pair. The features calculated are the difference in the percentage of every amino acid (20 features), the difference in molecular weight (1 feature), the difference in aromaticity index (1 feature), the difference in instability index (1 feature), the difference in the fraction of the total amino acids that are contained in the helix (1 feature), in the turn (1 feature) and in the sheet (1 feature), the difference in molar extinction coefficient, when the molar extinction coefficient is calculated assuming cysteines-reduced (1 feature), and when the molar extinction coefficient is calculated assuming cystines residues (Cys-Cys-bond, 1 feature), the difference in GRAVY (Grand Average of Hydropathy, 1 feature), and the difference in protein charge at pH = 7 (1 feature). A more thorough description of the features is presented in the Supplementary Table 2.

### Binding affinity dataset

The samples of protein–protein binding affinities were extracted from multiple experimental sources, including PDBbind v2020^122^, SKEMPI 2.0^123^, MPAD^124^, Binding Affinity Benchmark version 2^125^, and through literature mining. The data curation process started with a taxonomy filtering for Homo sapiens to retain only human PPI samples. To keep only the binary PPIs, we extracted data exclusively for dimeric complexes and removed entries where the binding affinity values were reported using units other than *K*_*d*_, *K*_*i*_, or Δ*G*. The samples with imprecise binding affinity values, such as ranges rather than exact values, were excluded to increase the consistency. Finally, the binding affinities were calculated using the equation ∆*G* = RT ln(*K*_*d*_) = RT ln(*K*_*i*_), applying the experimental temperature when specified, or 300 K if not provided. All redundant PPIs were eliminated as the last step of the dataset curation, resulting in a final dataset of 519 unique PPIs with experimentally determined binding affinities. These 519 PPIs were randomly split into training and test datasets, using an 80-20 training-test ratio, ending with 415 samples in the training set and 104 samples in the testing set.

### Model creation and evaluation

The first objective was the creation of a classification model for the prediction of PPIs. This classifier is a binary one, with the classes being “0” for negative and “1” for positive interactions, meaning that the models predict if there is going to be an interaction between the proposed two UniProt IDs, or not. The creation of the models was performed using two methodologies: (1) a BML approach based on state-of-the-art ML models (NB, Decision Tree (DT), SVM, Artificial Neural Network (ANN), RF, and XGB) using grid-search optimization for hyperparameter tuning, and (2) a multi-objective Pareto-based EOA approach. Both these models were used on the “PPI Dataset” described in the sections “PPI dataset building” and “Feature calculation.”

For the BML method, four ML models (NB, DT, SVM, and ANN) and two ensemble methods (RF and XGB) were evaluated. These models were trained on the feature-engineered dataset of positive and negative PPIs using specific parameters (detailed in Supplementary Table 3). Widely accepted tools and libraries were used, such as Python v3.10 and Scikit-Learn v1.1.2 for model implementation. The parameters used for optimizing the models are presented in the Supplementary Information section (Supplementary Table 3). Hyperparameter tuning was performed using a grid-search approach. This optimization process aimed to identify the best set of hyperparameters for each model to achieve optimal predictive performance. The grid-search parameters of each step in the model optimization process (imputation, feature selection, and outlier detection) are summarized in the Supplementary Information section (Supplementary Table 4). Values were scaled in the interval of [0,1] using a basic MinMaxScaler. Moreover, we tested three imputation methods: a simple approach that replaces missing values using the feature’s mean, an imputation for completing missing values using KNNs, and a multivariate imputer called Mice that estimates each feature from all the others^126^. Then, a feature selection method was applied to identify the best k-highest scores using the ANOVA *F*-value. Finally, two data outlier detection methods were used, i.e., an unsupervised outlier detection using the Local Outlier Factor^127^ and isolation forest^128^. Autoencoder architectures were tested for data augmentation. The process of data augmentation of the dataset consisted of a first training of the autoencoder with the training data of the dataset (epochs = 200, batch_size = 16), and later used to generate synthetic data samples. Several autoencoder architectures were studied (layers of size 56-37–28-37-56, 56-37–18-37-56, 56-37–14-37-56, 56-37–5-37-56). The new datasets consisted of the original dataset plus a synthetic dataset of the same size, thus duplicating the used data. To assess the predictive performance of the models, a stratified 10-fold CV was performed on the dataset to evaluate their robustness. The performance of the models was measured using several standard metrics, including accuracy, specificity, sensitivity, F1 score, F2 score, and ROC-AUC score.

The EOA method consists of a hybrid approach of heuristic optimization and nonlinear ML to develop classification models. Specifically, an ensemble dimensionality reduction technique was used that employed a heuristic multi-objective Pareto-based EOA to (a) identify the optimal feature subset to use as input to the classifiers, (b) select the most appropriate classifier among SVM and RF, and (c) select the optimal parameters for the classifier (e.g., C and gamma for SVM, the number of trees for RF). RF and SVM were chosen over DL and other ML methods, due to their robustness, interpretability, and applicability in a wide range of biological domains^129,130^. They have been used extensively for PPI classification and interaction site prediction^121,131–134^, both standalone and as a combination^135,136^. By utilizing them in the context of the multi-objective Pareto-based approach, we sought to optimize prediction performance, minimize the selected features, and simplify the classification model, aiming also to compare their performance to other DL and traditional ML approaches (BML method). The weights used for the optimization objectives were Selected Features Number Minimization 1, Accuracy (ACC) 10, F1 score 10, F2 score 1, Precision (PRC) 1, Recall (REC) 1, ROC-AUC (AUC) 1, Number of SVs or Trees Minimization 1, and Manhattan Distance 1. These weights allowed us to effectively address the imbalanced nature of our classification problem. The outcome is the generation of multiple models that exhibit similar performance concerning the user-defined objectives, which correspond to the Pareto set of optimal solutions that were represented as real number vectors. The evolutionary algorithm (EA) was applied to a population of 50 individuals, and the termination criteria were set to a maximum of 100 generations. Three different runs were conducted to deal with the stochastic nature of the algorithm, and the results presented are the average performance of these runs. Additional parameters of the EA were set to their default values (arithmetic crossover probability: 0, mutation probability: 0.01, two-point crossover probability: 0.9).

The training of the regression model was done using an extension of the EOA multi-objective algorithm for binary classification. It uses an ensemble EA to find the appropriate parameters for feature selection, the parameters of the regressors and the goals set. The regressors that are handled by the GA are the following: linear Support Vector Regressor (SVR), RBF SVR, RF Regressor, and Convolutional Neural Network. In the end, a group of different models is created that together have the best performance according to the goal weights set. In this case, the goals set are Goal Weight Significance for Feature (1), Negative MSE (10), MAE (1), Explained Variance (1), Model Complexity (1), RMSE (1), Correlation (1). Additional parameters of the EA were set to their default values (arithmetic crossover probability: 0, mutation probability: 0.01, two-point crossover probability: 0.9). The mean value predicted by the model group is set as the prediction value for each sample. For the training of the regression models, the EA was applied to a population of 50 individuals, and the termination criteria were set to a maximum of 1000 generations. Three different runs were conducted to deal with the stochastic nature of the algorithm.

For the evaluation of the classification models, a stratified 10-fold CV was performed in the training set. The metrics used in CV, training, and testing for the model evaluation are Accuracy, Specificity, Sensitivity, F1, F2, and ROC-AUC score. To evaluate the models of each run, two methods are implemented: the best model method and the ensemble method. For the best model method, model with the best weighted average of the performance metrics is used on the testing sets. On the other hand, the ensemble method is based on combining the Pareto front models into an ensemble classifier. Each of these models selects certain features. Then, for each sample, the models that have less than 50% missing values are combined to classify it using majority voting. The threshold that is applied for the classification models is that the Accuracy is higher than 80%. For the evaluation of the regression models, 10-fold CV was performed in the training dataset. We also used the same ensemble method that we used for the classifier to test the regression models, using a Correlation > 0.2 threshold to filter the models of the Pareto Front. The models that are not filtered out perform predictions on each sample’s endpoint, and then the final regression prediction is performed by averaging. The metrics used for the evaluation are: RMSE, RAE, RRSE, Spearman’s Rho, and *p* value of correlation.

The end goal is the testing of the classification and regression models on a dataset that encapsulates the potential TR interactome. The first step was the creation of a dataset consisting of the known TRs and their corresponding UniProt IDs. Information about the known TRs was retrieved from the work of Pallante et al.^6^ and Le et al.^137^. Based on these sources, we have considered the primary receptor candidates associated with each basic taste sensation. Specifically, our consideration includes the following: 25 bitter TRs from the TAS2Rs family of GPCRs, seven candidates for salty TRs encompassing subunits of the Epithelial Sodium Channel (α, β, γ, δ), Calcium homeostasis modulator 1 and 3 and Transient Receptor Potential Vanilloid 1 (TRPV1), the sweet receptor comprising the TAS1R2-TAS1R3 heterodimer, candidates for umami receptors (TAS1R1-TAS1R3, GRM1, GRM4, GPC6A, CASR, and LPAR5), and the sour receptor candidate (OTOP1). The table with these receptors, along with their respective UniProt IDs, is presented in the Supplementary Information section (Supplementary Table 5). These UniProt IDs are combined with all the known human UniProt IDs contained in the UniProt Database and are also combined with each other. By keeping only the reviewed entries, as of 21/12/2023, UniProt contained 20,482 IDs. The resulting data frame contains 816,518 combinations of TRs. For these combinations, the same features that were used for PPI and affinity datasets were again calculated.

### Molecular dynamics

MD technique was used to shed light on the interaction behavior between the best-interacting protein pairs predicted by our ML model. The selection of the PPI pair for the MD simulation was based on the following criteria: (1) Both interacting proteins must have an available PDB structure. (2) At least one protein’s PDB structure must not be derived from homology modeling. (3) To limit the computational power required for the simulation, we decided to consider PPIs composed of proteins with less than 350 amino acids. (4) At least one of the two proteins must have sufficient information regarding its binding site to facilitate a more precise docking procedure. In this connection, the protein pair that satisfied all the criteria was the TAS2R41-CHMP4A (Supplementary Table 6). In detail, the interacting pair is composed of the TAS2R41 (UniProt ID: P59536) bitter receptor and the CHMP4A (UniProt ID: Q9BY43) protein. The atomistic structure of the TAS2R41 bitter receptor was retrieved from the BitterDB^46,86^ database. Instead, the atomistic structure of the CHMP4A protein was collected from the PDB ID: 5MK1^73^. The rationale behind the selection of the CHMP4A protein segment GLU212-SER222 arises from its involvement in interactions with other proteins, as evidenced by two experimental PDB structures^73,74^. Therefore, it is reasonable to hypothesize that this specific CHMP4A protein section could be the main player in the interaction with TAS2R41. These two proteins were docked using HDOCKlite v1.1^89,138^, and the first 10 docked poses were selected as starting points for the simulations. In addition, the TAS2R41 bitter receptor was also studied in stand-alone conditions.

Since the bitter receptors belong to the superfamily of transmembrane G protein-coupled receptors^6^, all 10 starting poses, plus the stand-alone one, were inserted in a phospholipid bilayer. The membrane was composed of 200 POPC phospholipids in total, constructed and solvated according to the TIP3P^139^ water model using CHARMM-GUI^140,141^. All the systems were composed of around 82,000 particles, after the addition of sodium and chloride ions at a concentration of 0.15 M. The CHARMM36m^142^ force field was used to define phospholipid and protein topology through an all-atom approach. Each system was minimized using the steepest descent method. We then performed the equilibration procedure through one MD simulation of 250 ps under the NVT ensemble and four MD simulations of 250 ps, 500 ps, 1 ns, and 5 ns under the NPT ensemble. Phospholipid position restraints were applied during the first five MD equilibrations and gradually removed from 1000 kJ/mol·nm^2^ to 0 kJ/mol·nm^2^. Protein position restraints were also gradually removed from 4000 kJ/mol·nm^2^ to 200 kJ/mol·nm^2^. For the equilibration protocol, the v-rescaling^143^ temperature coupling algorithm with a time constant of 1.0 ps was applied to keep the temperature at 310 K. The c-rescale^144^ semi-isotropic pressure coupling algorithm with a reference pressure of 1 bar and a time constant of 5.0 ps was employed. Then, all systems were simulated for the production run in the NPT ensemble with 4 fs time steps^145^, using v-rescaling^143^ thermostat and c-rescale^144^ barostat, with a time constant of 1.0 ps and 5.0 ps, respectively. Each starting pose was simulated for 500 ns in 3 replicas, for a total of 16.5 μs of sampled time. Electrostatic interactions were calculated by applying the particle-mesh Ewald^146^ method, and van der Waals interactions^147^ were defined within a cutoff of 1.2 nm. Periodic boundary conditions were applied in all directions. Trajectories were collected every 20 ps, and the VMD^56^ package was employed to visually inspect the simulated systems. GROMACS 2023^148,149^ package was used for simulations and data analysis. The last 50 ns of the 500 ns production run of each simulation were considered for the analyses.

## Supplementary information

Supplementary information

## Data Availability

Open-source codes and scripts used for the analysis are publicly available at the following GitHub repository (https://github.com/harzav/TR_PPI_project).

## Code availability

Open-source codes and scripts used for the analysis are publicly available at the following GitHub repository (https://github.com/HarrisZavs/TR_PPI).
