# Supplementary material for: Unravelling the human taste receptor interactome: machine learning and molecular modelling insights into protein-protein interactions
Source: NPJ Sci Food. 2025 Jul 1;9:113. doi: 10.1038/s41538-025-00478-9 (PMC12217742; doi:10.1038/s41538-025-00478-9)
Supplement: Supplementary file 1 — Supplementary information [file 41538_2025_478_MOESM1_ESM.docx]

Supplementary Information of Manuscript

Unravelling the Human Taste Receptor Interactome: Machine Learning and Molecular Modelling Insights into Protein-Protein Interactions.

*Harry Zaverdas^1,8^†, Filip Stojceski^2^†, Rocío Romero-Zaliz^3^, Lampros Androutsos^1^, Pantelis Makrygiannis^1^, Lorenzo Pallante^4^, Vanessa Martos^5,9^, Gianvito Grasso^2^, Marco A. Deriu^4^, Konstantinos Theofilatos^6,*^ and Seferina Mavroudi^1,7,*^*

*^1^InSyBio PC, Patras, 265 04, Greece.*

*^2^Dalle Molle Institute for Artificial Intelligence USI-SUPSI, Polo universitario Lugano - Campus Est, Via la Santa, 6962, Lugano-Viganello, Switzerland.*

*^3^Research Center in Information and Communication Technologies (CITIC), Andalusian Research Institute on Data Science and Computational intelligence (DaSCI), Dept. of Computer Science and AI, University of Granada, Granada, Spain.*

*^4^PolitoBIOMedLab, Department of Mechanical and Aerospace Engineering, Politecnico di Torino, Torino, 10129, Italy.*

*^5^Department of Plant Physiology, University of Granada, Granada, Spain.*

*^6^*[*School of Cardiovascular and Metabolic Medicine & Sciences,*](https://www.kcl.ac.uk/scmms/) *King’s College London, London, UK*

*^7^Department of Nursing, School of Health Rehabilitation Sciences, University of Patras, 265 04 Patras, Greece.*

*^8^Department of Medicine, School of Health Sciences, University of Patras, 265 04 Patras, Greece.*

*^9^Institute of Biotechnology (IBT-UGR), University of Granada, Granada, Spain*

** Shared Senior Authorship, Corresponding Author: Dr. Konstantinos Theofilatos: konstantinos.theofilatos@kcl.ac.uk*

*† Joint First Authors*

# Supplementary Tables

*Supplementary Table 1: iRefinex filtering criteria.*

| **Filter** | **IRefindex Target Column** | **Value** |
| --- | --- | --- |
| **Uniprot entries-PPIs Only** | 'uidA', 'uidB' | Must contain ‘uniprotID’ |
| **Unique Interactions Only** | 'Checksum_Interaction' | Unique RIGID numbers |
| **Method of Interaction detection** | ‘method’ | tandem affinity purification, two hybrids |
| **Host Organism** | 'Host_organism_taxid' | "taxid: 9606" |
| **Number of participants in the interaction** | 'numParticipants' |  |

*Supplementary Table 2: Features Description.*

| **Features** | **N° of columns assigned** | **Column names** | **Description** | **Values** |
| --- | --- | --- | --- | --- |
| **Uniprot IDs of the Interacting Proteins** | 2 | uidA, uidB | Names of the two proteins in each PPI | String values |
| **NCBI RefSeq Accession Number IDs** | 2 | protein_accession_A, protein_accession_B | The respective NCBI IDs of the interacting proteins | String values, NaN |
| **PPI type** | 1 | PPI_type | Indicates if the PPI is positive or negative | 0,1 |
| **GO term similarity** | 3 | BP_similarity, MF_similarity, CC_similarity | The similarity of the proteins in the pair based on the similarity of their respective GO terms | Continuous Values from 0 to 1, NaN |
| **Existence of a Homologous Interacting Pair in other organisms** | 4 | Homologous in Mouse, ...Drosophila, ...Yeast, ...Ecoli | Existence of corresponding homologous PPIs in Mouse, yeast, Drosophila, E. coli | 0, 1, NaN |
| **Existence of the PPI in other Databases** | 4 | Exists in DIP?, ...in APID?, ... in BIOGRID?, ... in MINT? | Existence of PPI in APID, DIP, BIOGRID and MINT databases | 0, 1 |
| **Sequence Similarity** | 1 | Sequence_similarity | E- value of the sequences of the proteins in each PPI | E- value |
| **Domain Interactions** | 1 | pfam_interaction | Presence of known domain interactions between the proteins in each PPI pair | 0, 1, NaN |
| **Subcellular co-localization** | 1 | Subcellular Co-localization? | Subcellular co-localization of the two proteins in the pair in eukaryotic cells | 0, 1, NaN |
| **Gene expression profile similarity** | 15 | 0, 1, ...., 15 | the similarity of the two proteins in terms of their Gene Expression among fifteen NCBI GEO gene expression datasets | Spearman index ([-1, 1] float number) |
| **Amino acid difference** | 20 | A%, ...., G % | The absolute difference in the percentage of every amino acid between PP | Float numbers |
| **Molecular weight difference** | 1 | MW dif | The absolute difference in molecular weight between PP | Float numbers |
| **Aromaticity index difference** | 1 | Aromaticity dif | The absolute difference in aromaticity index difference between PP | Float numbers |
| **Instability index difference** | 1 | Instability dif | The absolute difference in instability index between PP | Float numbers |
| **Amino acid fraction difference** | 3 | helix_fraction_dif, turn_fraction_dif, sheet_fraction_dif | The difference in fraction of total amino acids that are contained in 3 areas:  The fraction of aa in helix, the fraction of aa in turn, the fraction of aa in sheet | Float numbers |
| **Molar extinction coefficient difference** | 2 | cys_reduced_dif, cys_residues_dif | The difference in molar extinction coefficient when:  (a) The molar extinction coefficient is calculated assuming cysteines(reduced) and  (b) The molar extinction coefficient is calculated assuming cystines residues (Cys-Cys-bond) | Integers |
| **GRAVY (Grand Average of Hydropathy) difference** | 1 | gravy_dif | The absolute difference in GRAVY index between PP | Float numbers |
| **pH charge difference** | 1 | ph7_charge_dif | The absolute difference in the protein charge when pH= 7 | Float numbers |
| **RNA expression profile similarity** | 2 | GSE227375_spearman, GSE228702_spearman | The similarity between each protein in the pair, in terms of their RNA expression profiles (NCBI GEO GSE227375 and GSE228702 datasets) | Spearman index ([-1, 1] float number) |

*Supplementary Table 3: Parameters used for the Benchmark ML methods.*

| **ML method** | **Scikit-Learn parameters** |
| --- | --- |
| **Näive Bayes (NB)** | Gaussian Naive Bayes  var_smoothing=1e-09 |
| **Decision Tree (DT)** | criterion='gini', splitter='best', max_depth=5, min_samples_split=2, min_samples_leaf=1, min_weight_fraction_leaf=0.0, max_features=None, max_leaf_nodes=None, min_impurity_decrease=0.0, class_weight=None, ccp_alpha=0.0 |
| **Support Vector Machine (SVM)** | C=1.0, kernel='rbf', degree=3, gamma='scale', coef0=0.0, shrinking=True, probability=False, tol=0.001, cache_size=200, class_weight=None, max_iter=-1, decision_function_shape='ovr', break_ties=False |
| **Artificial Neural Network (ANN)** | MLPClassifier with two hidden layers of 30 neurons and 10 neurons respectively.  solver=‘sgd', alpha=1e-5, max_iter=5000, activation='relu', batch_size='auto', learning_rate='constant', learning_rate_init=0.001, power_t=0.5, max_iter=200, shuffle=True, tol=0.0001, warm_start=False, momentum=0.9, nesterovs_momentum=True, early_stopping=False, validation_fraction=0.1, beta_1=0.9, beta_2=0.999, epsilon=1e-08, n_iter_no_change=10, max_fun=15000 |
| **Random Forest (RF)** | n_estimators=5, criterion='gini', max_depth=None, min_samples_split=2, min_samples_leaf=1, min_weight_fraction_leaf=0.0, max_features='sqrt', max_leaf_nodes=None, min_impurity_decrease=0.0, bootstrap=True, oob_score=False, n_jobs=None, warm_start=False, class_weight=None, ccp_alpha=0.0, max_samples=None |
| **XGBoost (XGB)** | base_score=0.5, colsample_bylevel=1, colsample_bytree=1, gamma=0, learning_rate=0.1, max_delta_step=0, max_depth=5, min_child_weight=1, missing=None, n_estimators=100, nthread=-1, objective='binary:logistic', reg_alpha=0, reg_lambda=1, scale_pos_weight=1, subsample=1, tree_method='hist', eta=0.3 |

*Supplementary Table 4: Hyperparameters used for tuning Benchmark ML models.*

| **Parameter type** | **Parameter** | **Optional arguments** |
| --- | --- | --- |
| **Imputation** | None |  |
|  | Simple | strategy='mean', fill_value=None, keep_empty_features=False |
|  | KNN | n_neighbors=5, weights='uniform', metric=‘nan_euclidean’ |
|  | Mice | estimator=‘bayesian ridge’, sample_posterior=False, max_iter=10, tol=0.001, n_nearest_features=None, initial_strategy='mean', fill_value=None, imputation_order='ascending', skip_complete=False, min_value=-inf, max_value=inf, keep_empty_features=False |
| **Feature selection** | None |  |
|  | Best k | k in [5, 10, 15, 20, 25, 30] |
| **Outlier detection** | None |  |
|  | Local | n_neighbors=20, algorithm='auto', leaf_size=30, metric='minkowski', p=2, contamination='auto', novelty=False |
|  | Isolation Forest | number of estimators=10, max_samples='auto', contamination='auto', max_features=1.0, bootstrap=False, warm_start=True |

*Supplementary Table 5: List of known taste receptors, their Uniprot IDs and names and the corresponding taste perception they are involved.*

| uid | name | taste |
| --- | --- | --- |
| Q8TE23 | TS1R2_HUMAN | sweet |
| Q7RTX0 | TS1R3_HUMAN | sweet/umami |
| Q7RTX1 | TS1R1_HUMAN | umami |
| Q13255 | GRM1_HUMAN | umami |
| Q14833 | GRM4_HUMAN | umami |
| Q5T6X5 | GPC6A_HUMAN | umami |
| P41180 | CASR_HUMAN | umami |
| Q9H1C0 | LPAR5_HUMAN | umami |
| Q9NYW0 | T2R10_HUMAN | bitter |
| Q9NYV8 | T2R14_HUMAN | bitter |
| P59540 | T2R46_HUMAN | bitter |
| Q7RTR8 | T2R42_HUMAN | bitter |
| P59539 | T2R45_HUMAN | bitter |
| P59542 | T2R19_HUMAN | bitter |
| P59551 | T2R60_HUMAN | bitter |
| Q9NYW7 | TA2R1_HUMAN | bitter |
| Q9NYV9 | T2R13_HUMAN | bitter |
| Q9NYV7 | T2R16_HUMAN | bitter |
| Q9NYW6 | TA2R3_HUMAN | bitter |
| P59533 | T2R38_HUMAN | bitter |
| P59534 | T2R39_HUMAN | bitter |
| Q9NYW5 | TA2R4_HUMAN | bitter |
| P59535 | T2R40_HUMAN | bitter |
| P59536 | T2R41_HUMAN | bitter |
| P59537 | T2R43_HUMAN | bitter |
| P59538 | T2R31_HUMAN | bitter |
| P59541 | T2R30_HUMAN | bitter |
| P59543 | T2R20_HUMAN/TAS2R20, TAS2R49 | bitter |
| [Q9NYW4](https://www.uniprot.org/uniprotkb/Q9NYW4/entry) | TA2R5_HUMAN | bitter |
| [P59544](https://www.uniprot.org/uniprotkb/P59544/entry) | T2R50_HUMAN | bitter |
| [Q9NYW3](https://www.uniprot.org/uniprotkb/Q9NYW3/entry) | TA2R7_HUMAN | bitter |
| [Q9NYW2](https://www.uniprot.org/uniprotkb/Q9NYW2/entry) | TA2R8_HUMAN | bitter |
| [Q9NYW1](https://www.uniprot.org/uniprotkb/Q9NYW1/entry) | TA2R9_HUMAN | bitter |
| Q8IU99 | CAHM1_HUMAN | salty |
| Q86XJ0 | CAHM3_HUMAN | salty |
| P37088 | SCNNA_HUMAN/ENaC Sodium channel epithelial 1 subunit alpha | salty |
| P51168 | SCNNB_HUMAN/ ENaC Sodium channel epithelial 1 subunit beta | salty |
| P51172 | SCNND_HUMAN/ ENaC Sodium channel epithelial 1 subunit delta | salty |
| P51170 | SCNNG_HUMAN/ ENaC Sodium channel epithelial 1 subunit gamma | salty |
| [Q7RTM1](https://www.uniprot.org/uniprotkb/Q7RTM1/entry) | OTOP1_HUMAN | sour |
| Q8NER1 | TRPV1_HUMAN | salty |

*Supplementary Table 6: Top 20 TR interactions overall. TR interaction (UID): Taste Receptor Interaction’s Uniprot IDs, Gene1/ Gene2: Taste Receptor Interaction’s Gene Names, Predicted Classes: Indication of the positive class prediction (1), irefindex_check: 1 if the interaction has been recorded in iRefIndex, 0 if not, Probability Score: The classifier’s prediction probability score, Aff_predictions: The binding strength (affinity) predictions of the regressor, Mean_prob_aff: The mean of classifier’s prediction probability score and the predicted binding strength.*

| TR interaction (UID) | Gene1 | Gene2 | Predicted Classes | Probability Score | Aff_predictions | Mean_prob_aff | irefindex_check |
| --- | --- | --- | --- | --- | --- | --- | --- |
| P0C0E4-P59551 | RAB40AL | TAS2R60 | 1 | 0.99 | 0.41 | 0.70 | 0 |
| A1A580-Q7RTM1 | KRTAP23-1 | OTOP1 | 1 | 0.98 | 0.41 | 0.69 | 0 |
| P37088-P51170 | SCNN1A | SCNN1G | 1 | 0.98 | 0.40 | 0.69 | 0 |
| P05771-P41180 | PRKCB | CASR | 1 | 0.98 | 0.40 | 0.69 | 1 |
| P59540-Q9NY47 | TAS2R46 | CACNA2D2 | 1 | 0.97 | 0.40 | 0.68 | 0 |
| Q92556-Q9NYW3 | ELMO1 | TAS2R7 | 1 | 0.98 | 0.39 | 0.68 | 0 |
| P49753-Q13255Β | ACOT2 | GRM1 | 1 | 0.97 | 0.40 | 0.68 | 0 |
| Q8WYK1-Q9NYW5 | CNTNAP5 | TAS2R4 | 1 | 0.97 | 0.40 | 0.68 | 0 |
| P37088-Q9NRS4 | SCNN1A | TMPRSS4 | 1 | 0.98 | 0.39 | 0.68 | 0 |
| P21439-Q7RTM1 | ABCB4 | OTOP1 | 1 | 0.97 | 0.40 | 0.68 | 0 |
| Q02763-Q9NYW2 | TEK | TAS2R8 | 1 | 0.97 | 0.40 | 0.68 | 0 |
| A8MSI8-P59535 | LYRM9 | TAS2R40 | 1 | 0.96 | 0.40 | 0.68 | 0 |
| P59551-Q7L5N7 | TAS2R60 | LPCAT2 | 1 | 0.97 | 0.40 | 0.68 | 0 |
| O75030-P59536 | MITF | TAS2R41 | 1 | 0.95 | 0.40 | 0.68 | 0 |
| P55291-P59534 | CDH15 | TAS2R39 | 1 | 0.92 | 0.43 | 0.67 | 0 |
| P59536-Q9BY43 | TAS2R41 | CHMP4A | 1 | 0.94 | 0.40 | 0.67 | 1 |
| P59539-Q9NQS1 | TAS2R45 | AVEN | 1 | 0.95 | 0.40 | 0.67 | 0 |
| P59540-Q7L014 | TAS2R46 | DDX46 | 1 | 0.93 | 0.41 | 0.67 | 0 |
| P15151-P59542 | PVR | TAS2R19 | 1 | 0.94 | 0.40 | 0.67 | 1 |
| Q9BQ51-Q9NYW0 | PDCD1LG2 | TAS2R10 | 1 | 0.93 | 0.40 | 0.67 | 1 |

*Supplementary Table 7: Top 15 TR interactions including UMAMI receptors. TR interaction (UID): Taste Receptor Interaction’s Uniprot IDs, Gene1/ Gene2: Taste Receptor Interaction’s Gene Names, Predicted Classes: Indication of the positive class prediction (1), irefindex_check: 1 if the interaction has been recorded in iRefIndex, 0 if not, Probability Score: The classifier’s prediction probability score, Aff_predictions: The binding strength (affinity) predictions of the regressor, Mean_prob_aff: The mean of classifier’s prediction probability score and the predicted binding strength.*

| TR interaction (UID) | Gene1 | Gene2 | Predicted Classes | Probability Score | Aff_predictions | Mean_prob_aff | irefindex_check |
| --- | --- | --- | --- | --- | --- | --- | --- |
| P05771-P41180 | PRKCB | CASR | 1 | 0.98 | 0.40 | 0.69 | 1 |
| P49753-Q13255Β | ACOT2 | GRM1 | 1 | 0.97 | 0.40 | 0.68 | 0 |
| Q13255Β -Q8WV37 | GRM1 | ZNF480 | 1 | 0.91 | 0.40 | 0.66 | 0 |
| Q12983-Q7RTX1 | BNIP3 | TAS1R1 | 1 | 0.91 | 0.40 | 0.66 | 0 |
| P35712-Q7RTX0 | SOX6 | TAS1R3 | 1 | 0.88 | 0.42 | 0.65 | 0 |
| P42338-Q7RTX0 | PIK3CB | TAS1R3 | 1 | 0.87 | 0.41 | 0.64 | 0 |
| P54296-Q7RTX1 | MYOM2 | TAS1R1 | 1 | 0.83 | 0.41 | 0.62 | 0 |
| P41180-P55259 | CASR | GP2 | 1 | 0.83 | 0.40 | 0.62 | 0 |
| Q5T6X5-Q9H2C8 | GPRC6A | OR51V1 | 1 | 0.82 | 0.40 | 0.61 | 0 |
| Q7RTX1-Q8ND94 | TAS1R1 | LRRN4CL | 1 | 0.80 | 0.40 | 0.60 | 0 |
| P41180-Q00056 | CASR | HOXA4 | 1 | 0.80 | 0.40 | 0.60 | 0 |
| O95758-P41180 | PTBP3 | CASR | 1 | 0.78 | 0.40 | 0.59 | 0 |
| Q14833Β -Q86T20 | GRM4 | SMIM29 | 1 | 0.76 | 0.40 | 0.58 | 0 |
| Q14833Β -Q5T1C6 | GRM4 | THEM4 | 1 | 0.74 | 0.40 | 0.57 | 0 |
| Q9H1C0Β -Q9NQ86 | LPAR5 | TRIM36 | 1 | 0.67 | 0.40 | 0.53 | 0 |

*Supplementary Table 8: Top 15 TR interactions including SALTY receptors. TR interaction (UID): Taste Receptor Interaction’s Uniprot IDs, Gene1/ Gene2: Taste Receptor Interaction’s Gene Names, Predicted Classes: Indication of the positive class prediction (1), irefindex_check: 1 if the interaction has been recorded in iRefIndex, 0 if not, Probability Score: The classifier’s prediction probability score, Aff_predictions: The binding strength (affinity) predictions of the regressor, Mean_prob_aff: The mean of classifier’s prediction probability score and the predicted binding strength.*

| TR interaction (UID) | Gene1 | Gene2 | Predicted Classes | Probability Score | Aff_predictions | Mean_prob_aff | irefindex_check |
| --- | --- | --- | --- | --- | --- | --- | --- |
| P37088-P51170 | SCNN1A | SCNN1G | 1 | 0.98 | 0.40 | 0.69 | 0 |
| P37088-Q9NRS4 | SCNN1A | TMPRSS4 | 1 | 0.98 | 0.39 | 0.68 | 0 |
| P51168-Q8NBJ7 | SCNN1B | SUMF2 | 1 | 0.93 | 0.40 | 0.66 | 0 |
| P51168-Q5T3J3 | SCNN1B | LRIF1 | 1 | 0.92 | 0.40 | 0.66 | 0 |
| P37088-Q9Y6I3 | SCNN1A | EPN1 | 1 | 0.91 | 0.40 | 0.66 | 0 |
| P51172-Q2PZI1 | SCNN1D | DPY19L1 | 1 | 0.89 | 0.40 | 0.65 | 1 |
| O95661-P51172 | DIRAS3 | SCNN1D | 1 | 0.89 | 0.41 | 0.65 | 0 |
| P48730-Q8IU99 | CSNK1D | CALHM1 | 1 | 0.88 | 0.40 | 0.64 | 0 |
| Q86XJ0-Q9H0T7 | CALHM3 | RAB17 | 1 | 0.88 | 0.40 | 0.64 | 0 |
| P37088-Q9NWH7 | SCNN1A | SPATA6 | 1 | 0.87 | 0.40 | 0.64 | 0 |
| O75604-P51170 | USP2 | SCNN1G | 1 | 0.87 | 0.39 | 0.63 | 0 |
| Q8IU99-Q8WWF1 | CALHM1 | C1orf54 | 1 | 0.87 | 0.40 | 0.63 | 0 |
| Q86XJ0-Q9NV70 | CALHM3 | EXOC1 | 1 | 0.87 | 0.39 | 0.63 | 0 |
| Q8IU99-Q8IU99 | CALHM1 | CALHM1 | 1 | 0.84 | 0.42 | 0.63 | 1 |
| P37088-P37088 | SCNN1A | SCNN1A | 1 | 0.84 | 0.42 | 0.63 | 1 |

*Supplementary Table 9: Top 15 TR interactions including SOUR receptors. TR interaction (UID): Taste Receptor Interaction’s Uniprot IDs, Gene1/ Gene2: Taste Receptor Interaction’s Gene Names, Predicted Classes: Indication of the positive class prediction (1), irefindex_check: 1 if the interaction has been recorded in iRefIndex, 0 if not, Probability Score: The classifier’s prediction probability score, Aff_predictions: The binding strength (affinity) predictions of the regressor, Mean_prob_aff: The mean of classifier’s prediction probability score and the predicted binding strength.*

| TR interaction (UID) | Gene1 | Gene2 | Predicted Classes | Probability Score | Aff_predictions | Mean_prob_aff | irefindex_check |
| --- | --- | --- | --- | --- | --- | --- | --- |
| A1A580-Q7RTM1 | KRTAP23-1 | OTOP1 | 1 | 0.98 | 0.41 | 0.69 | 0 |
| P21439-Q7RTM1 | ABCB4 | OTOP1 | 1 | 0.97 | 0.40 | 0.68 | 0 |
| Q00587-Q7RTM1 | CDC42EP1 | OTOP1 | 1 | 0.85 | 0.41 | 0.63 | 0 |
| Q7RTM1-Q9BZF1 | OTOP1 | OSBPL8 | 1 | 0.78 | 0.41 | 0.59 | 0 |
| Q7RTM1-Q9NUC0 | OTOP1 | SERTAD4 | 1 | 0.63 | 0.41 | 0.52 | 0 |
| Q2TAP0-Q7RTM1 | GOLGA7B | OTOP1 | 1 | 0.55 | 0.42 | 0.49 | 0 |
| A5LHX3-Q7RTM1 | PSMB11 | OTOP1 | 1 | 0.55 | 0.42 | 0.49 | 0 |
| Q7RTM1-Q96LU7 | OTOP1 | MYRFL | 1 | 0.55 | 0.42 | 0.49 | 0 |
| Q7RTM1-Q8TED1 | OTOP1 | GPX8 | 1 | 0.55 | 0.42 | 0.49 | 0 |
| P43681-Q7RTM1 | CHRNA4 | OTOP1 | 1 | 0.55 | 0.42 | 0.49 | 0 |
| Q6UW78-Q7RTM1 | UQCC3 | OTOP1 | 1 | 0.55 | 0.42 | 0.49 | 0 |
| A0A0J9YWL9-Q7RTM1 | TEX13C | OTOP1 | 1 | 0.55 | 0.42 | 0.49 | 0 |
| Q7RTM1-Q9NQ69 | OTOP1 | LHX9 | 1 | 0.57 | 0.40 | 0.49 | 0 |
| Q6UXP9-Q7RTM1 | Q6UXP9 | OTOP1 | 1 | 0.55 | 0.42 | 0.48 | 0 |
| Q6P5R6-Q7RTM1 | RPL22L1 | OTOP1 | 1 | 0.55 | 0.42 | 0.48 | 0 |

*Supplementary Table 10: Top 15 TR interactions including BITTER receptors. TR interaction (UID): Taste Receptor Interaction’s Uniprot IDs, Gene1/ Gene2: Taste Receptor Interaction’s Gene Names, Predicted Classes: Indication of the positive class prediction (1), irefindex_check: 1 if the interaction has been recorded in iRefIndex, 0 if not, Probability Score: The classifier’s prediction probability score, Aff_predictions: The binding strength (affinity) predictions of the regressor, Mean_prob_aff: The mean of classifier’s prediction probability score and the predicted binding strength.*

| TR interaction (UID) | Gene1 | Gene2 | Predicted Classes | Probability Score | Aff_predictions | Mean_prob_aff | irefindex_check |
| --- | --- | --- | --- | --- | --- | --- | --- |
| P0C0E4-P59551 | RAB40AL | TAS2R60 | 1 | 0.99 | 0.41 | 0.70 | 0 |
| P59540-Q9NY47 | TAS2R46 | CACNA2D2 | 1 | 0.97 | 0.40 | 0.68 | 0 |
| Q92556-Q9NYW3 | ELMO1 | TAS2R7 | 1 | 0.98 | 0.39 | 0.68 | 0 |
| Q8WYK1-Q9NYW5 | CNTNAP5 | TAS2R4 | 1 | 0.97 | 0.40 | 0.68 | 0 |
| Q02763-Q9NYW2 | TEK | TAS2R8 | 1 | 0.97 | 0.40 | 0.68 | 0 |
| A8MSI8-P59535 | LYRM9 | TAS2R40 | 1 | 0.96 | 0.40 | 0.68 | 0 |
| P59551-Q7L5N7 | TAS2R60 | LPCAT2 | 1 | 0.97 | 0.40 | 0.68 | 0 |
| O75030-P59536 | MITF | TAS2R41 | 1 | 0.95 | 0.40 | 0.68 | 0 |
| P55291-P59534 | CDH15 | TAS2R39 | 1 | 0.92 | 0.43 | 0.67 | 0 |
| P59536-Q9BY43 | TAS2R41 | CHMP4A | 1 | 0.94 | 0.40 | 0.67 | 1 |
| P59539-Q9NQS1 | TAS2R45 | AVEN | 1 | 0.95 | 0.40 | 0.67 | 0 |
| P59540-Q7L014 | TAS2R46 | DDX46 | 1 | 0.93 | 0.41 | 0.67 | 0 |
| P15151-P59542 | PVR | TAS2R19 | 1 | 0.94 | 0.40 | 0.67 | 1 |
| Q9BQ51-Q9NYW0 | PDCD1LG2 | TAS2R10 | 1 | 0.93 | 0.40 | 0.67 | 1 |
| Q15437-Q9NYV7 | SEC23B | TAS2R16 | 1 | 0.93 | 0.40 | 0.67 | 0 |

*Supplementary Table 11: Top 15 TR interactions including SWEET receptors. TR interaction (UID): Taste Receptor Interaction’s Uniprot IDs, Gene1/ Gene2: Taste Receptor Interaction’s Gene Names, Predicted Classes: Indication of the positive class prediction (1), irefindex_check: 1 if the interaction has been recorded in iRefIndex, 0 if not, Probability Score: The classifier’s prediction probability score, Aff_predictions: The binding strength(affinity) predictions of the regressor, Mean_prob_aff: The mean of classifier’s prediction probability score and the predicted binding strength.*

| TR interaction (UID) | Gene1 | Gene2 | Predicted Classes | Probability Score | Aff_predictions | Mean_prob_aff | irefinedx_check |
| --- | --- | --- | --- | --- | --- | --- | --- |
| P35712-Q7RTX0 | SOX6 | TAS1R3 | 1 | 0.88 | 0.42 | 0.65 | 0 |
| P42338-Q7RTX0 | PIK3CB | TAS1R3 | 1 | 0.87 | 0.41 | 0.64 | 0 |
| Q8TE23Β -Q9NP92 | TAS1R2 | MRPS30 | 1 | 0.80 | 0.41 | 0.60 | 0 |
| Q8TE23Β -Q9H171 | TAS1R2 | ZBP1 | 1 | 0.58 | 0.40 | 0.49 | 0 |
| Q7RTX0-Q96NU0 | TAS1R3 | CNTNAP3B | 1 | 0.55 | 0.42 | 0.49 | 0 |
| Q8TE23Β -Q99707 | TAS1R2 | MTR | 1 | 0.55 | 0.42 | 0.49 | 0 |
| Q8TE23Β -Q9UKU9 | TAS1R2 | ANGPTL2 | 1 | 0.55 | 0.42 | 0.49 | 0 |
| Q8TE23Β -Q9Y4L5 | TAS1R2 | RNF115 | 1 | 0.55 | 0.42 | 0.49 | 0 |
| Q8TE23Β -Q9BZY9 | TAS1R2 | TRIM31 | 1 | 0.55 | 0.42 | 0.49 | 0 |
| Q8TE23Β -Q96PL2 | TAS1R2 | TECTB | 1 | 0.55 | 0.42 | 0.49 | 0 |
| Q8TE23Β -Q96EX2 | TAS1R2 | RNFT2 | 1 | 0.55 | 0.42 | 0.49 | 0 |
| Q8TE23Β -Q9HC44 | TAS1R2 | GPBP1L1 | 1 | 0.55 | 0.42 | 0.49 | 0 |
| P06493-Q7RTX0 | CDK1 | TAS1R3 | 1 | 0.55 | 0.42 | 0.49 | 0 |
| P35236-Q7RTX0 | PTPN7 | TAS1R3 | 1 | 0.55 | 0.42 | 0.49 | 0 |
| A0MZ66-Q8TE23Β | SHTN1 | TAS1R2 | 1 | 0.55 | 0.42 | 0.49 | 0 |

*Supplementary Table 12: Features selected by the best scoring models in classification and regression analyses.*

| Analysis | Features Selected by Best Scoring Model in each Analysis |
| --- | --- |
| Classification | 'MF_similarity', 'CC_similarity', 'Exists in MINT?', 'Exists in APID?', 'Exists in BIOGRID?', 'pfam_interaction', 'A %', 'I %', 'V %', 'S %', 'C %', 'W %', 'R %', 'cys_reduced_dif' |
| Regression | ‘A %’, ‘L %’,‘F %’, ‘I %’, ‘M %’, ‘V %’, ‘S %’, ‘P %’, ‘T %’,‘Y %’, ‘H %’, ‘Q %’, ‘N %’, ‘K%’, ‘D %’, ‘E %’, ‘C %’, ‘W %’, ‘R %’, ‘G %’, ‘MW dif’, ‘Aromaticity dif’, ‘Instability dif’, ‘helix_fraction_dif’, ‘turn_fraction_dif’, ‘sheet_fraction_dif’, ‘cys_reduced_dif’, ‘cys_residues_dif’, ‘gravy_dif’, ‘ph7_charge_dif’, ‘BP_similarity’, ‘MF_similarity’, ‘CC_similarity’, ‘Exists in MINT?’, ‘Exists in DIP?’, ‘Exists in APID?’, ‘Exists in BIOGRID?’, ‘Homologous in Mouse’, ‘Homologous in Drosophila’, ‘pfam_interaction’, ‘0’, ‘1’, ‘2’, ‘3’, ‘4’, ‘7’, ‘8’, ‘10’, Subcellular Co-localization?’, ‘5’, ‘6’, ‘9’, ‘11’, ‘12’, ‘13’, ‘14’, ‘GSE227375_spearman’, ‘GSE228702_spearman’ |

# Supplementary Figures


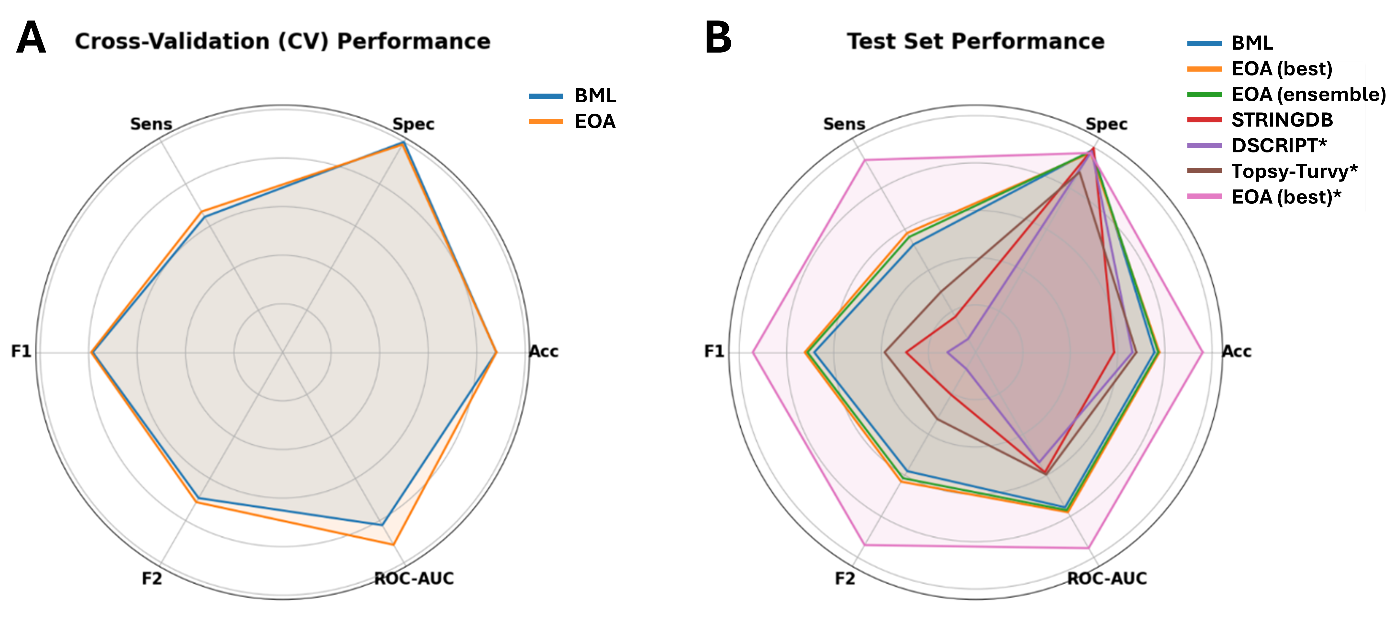


*Supplementary Figure 1: Spiderplots visualizing the CV and testing performance of Binary Classifier using benchmark machine-learning techniques (ML) and the Multi-objective Pareto-based evolutionary optimization algorithm (EOA). A) Cross-validation (CV) performance metrics are displayed for BML (blue) and EOA (orange) methodologies. B) Test set performance of BML and EOA methodologies in comparison with multiple models. The methods tested were: BML in blue, EOA (best) in orange, EOA (ensemble) in green, STRINGDB in red, D-SCRIPT* in purple, Topsy-Turvy* in brown, and EOA (best)* in pink. Asterisk (*) indicates models evaluated on a reduced version of the test dataset (34,362 PPIs, negative instances: 22,644, positive instances: 11,718). ‘EOA (ensemble)’ testing references to the ensemble testing method based on majority voting of the Pareto Front models, while EOA (best) references to the Pareto Front best model testing method. Performance is evaluated using Accuracy (Acc), Sensitivity (Sens), Specificity (Spec), F1 score, F2 score, and ROC-AUC score.*


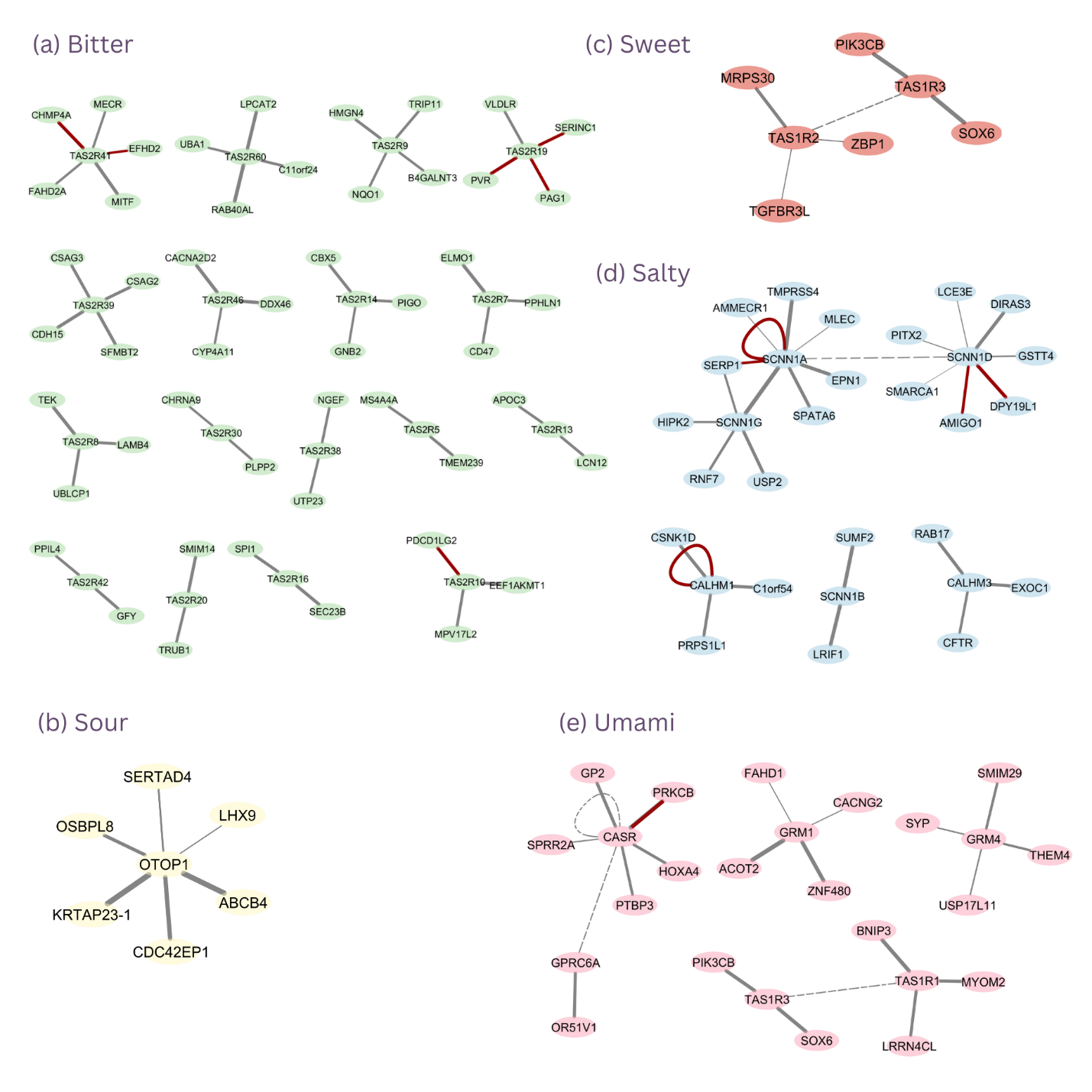


*Supplementary Figure 2: Interaction network of the top scoring interactions involving (a) bitter, (b) sour, (c) sweet, (d) salty and (e) umami receptors. Red lines denote that the interaction was predicted by the classification models, while also is recorded in iRefindex. Dotted lines denote that the interaction was not predicted by the classification models but is recorded in iRefindex. Edge width is analogous to the mean of the classifier’s probability score and the predicted binding strength of the interaction.*


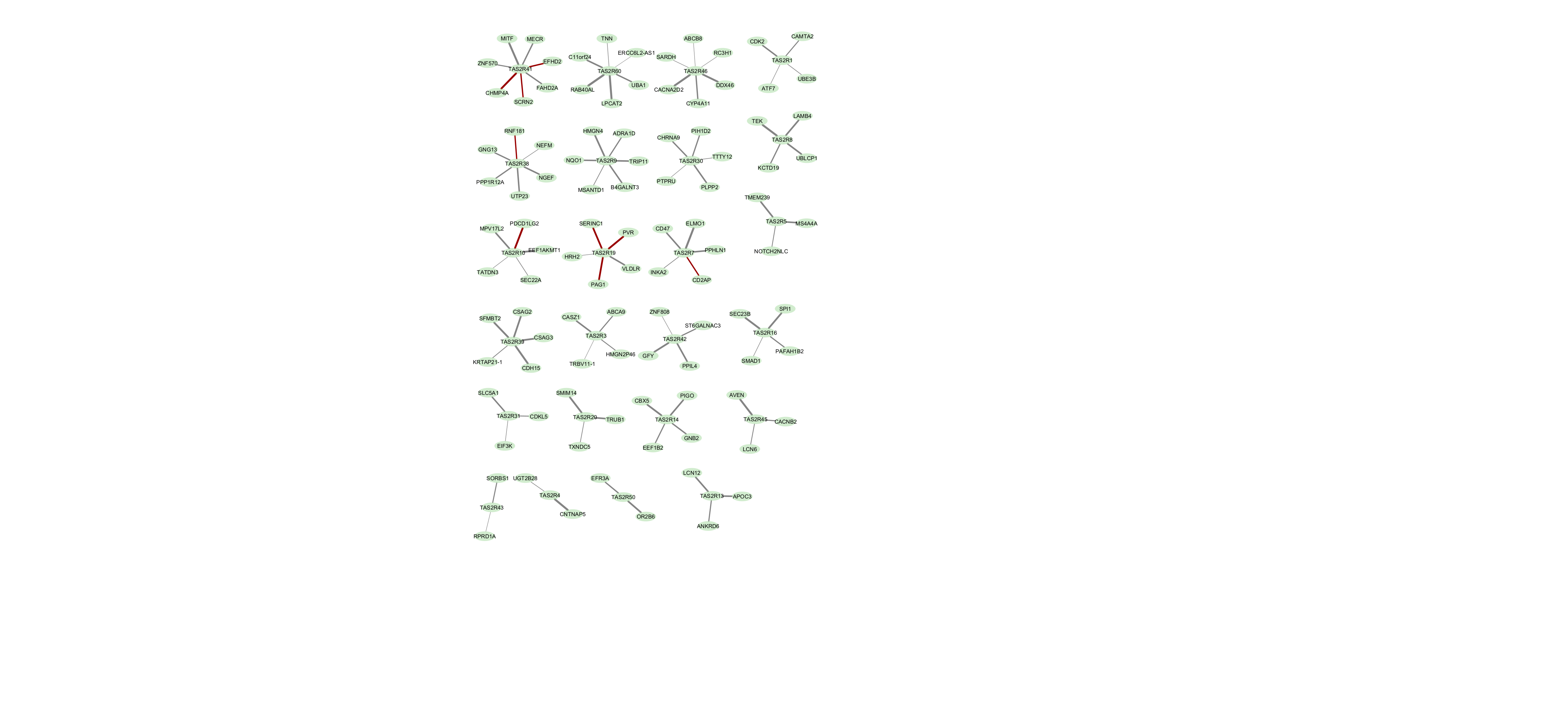


*Supplementary Figure 3: Full interaction network (no extra filter applied) of the top scoring interactions involving bitter receptors. Red lines denote that the interaction was predicted by the classification models, while also is recorded in iRefindex. Dotted lines denote that the interaction was not predicted by the classification models but is recorded in iRefindex. Edge width is analogous to the mean of the classifier’s probability score and the predicted binding strength of the interaction.*


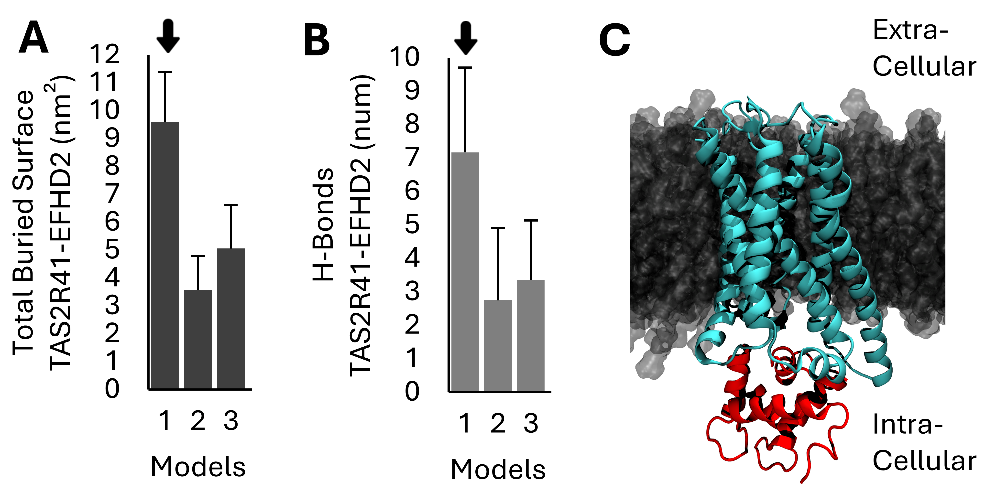


Supplementary Figure 4: **A**) Total buried surface (TBS) analysis between TAS2R41 bitter receptor and EFHD2 protein of the 3 models. The highest total buried surface value between the 3 models is highlighted by the black arrow. **B**) H-Bonds analysis between TAS2R41 bitter receptor and EFHD2 protein of the 3 models. The highest H-Bonds value between the 3 models is highlighted by the black arrow. **C**) Qualitative representation of the first model inside the POPC membrane. In green, it is shown the TAS2R41 bitter receptor, while in red it is shown the EFHD2 protein.
